# Study and Evaluation of Equivalent Conductivities of [SiO(OH)_3_]^−^ and [SiO_2_(OH)_2_]^2−^ in NaOH-Na_2_SiO_3_-H_2_O Solutions at 277.85 K to 308.45 K

**DOI:** 10.3390/ma18132996

**Published:** 2025-06-24

**Authors:** Kai Yang, Guang Ye, Geert De Schutter

**Affiliations:** 1 Department of Structural Engineering and Building Materials, Ghent University, B-9052 Ghent, Belgium; kai.yang@ugent.be; 2 Department of Materials and Environment (Microlab), Delft University of Technology, 2628 CN Delft, The Netherlands; g.ye@tudelft.nl

**Keywords:** equivalent conductivity, electrochemical impedance spectroscopy, mixed electrolytes, silicates, regression analysis

## Abstract

The equivalent conductivities of two aqueous silicate species, ${\left[\mathrm{SiO}{\left(\mathrm{OH}\right)}_{3}\right]}^{-}$ and ${\left[\mathrm{Si}O_{2}{\left(\mathrm{OH}\right)}_{2}\right]}^{2-}$, are fundamental to understanding many physico-chemical phenomena of silicate materials in electrolyte solutions. These phenomena include diffusion, adsorption, and phase transformations. But significant inconsistencies have been presented in published equivalent conductivities of the two silicate aqueous ions. Also, little work has so far been undertaken to discuss how aspects, such as temperature and solution composition, may influence electrolytic conductivity of silicate aqueous solutions. This work presents the equivalent conductivities of the two silicate species, measured with electrochemical impedance spectroscopy (EIS) from 277.85 K to 308.45 K. A conductivity model for mixed electrolytes of high alkaline was first established. This model was then verified with the electrolyte conductivities of $\mathrm{NaOH}\text{-}H_{2}O$ solutions and $\mathrm{NaOH}\text{-}Na_{2}CO_{3}\text{-}H_{2}O$ solutions. Next, the equivalent conductivities of ${\left[\mathrm{SiO}{\left(\mathrm{OH}\right)}_{3}\right]}^{-}$ and ${\left[\mathrm{Si}O_{2}{\left(\mathrm{OH}\right)}_{2}\right]}^{2-}$, were calculated by solving the overdetermined equation groups for different temperatures, based on electrolyte conductivities of $\mathrm{NaOH}\text{-}Na_{2}\mathrm{Si}O_{3}\text{-}H_{2}O$ solutions. The accuracy of both calculations and measurements are examined in depth from various viewpoints. This work presents essential inputs for quantitatively understanding multiple physico-chemical properties of silicate materials in electrolyte solutions.

## 1. Introduction

Physico-chemical properties of silicate aqueous solution have become multidisciplinary interests in recent years. One of the main reasons is that the requirement of global sustainability urges scientists to fundamentally understand concrete. Concrete, as the highest consumed material on earth besides water, is responsible for 8% of worldwide carbon emissions [1]. The main binding phase in concrete is calcium silicate hydrates (C-S-H) [2]. Thus, the mechanism controlling the phase transformations of C-S-H in aqueous solution has been the crucial research subject to develop a bottom-top technology for sustainability. Besides surface hydroxylation, these sophisticated phase transformations in solutions start with the clustering of monomeric silicate species, including ${\left[\mathrm{SiO}{\left(\mathrm{OH}\right)}_{3}\right]}^{-}$ and ${\left[\mathrm{Si}O_{2}{\left(\mathrm{OH}\right)}_{2}\right]}^{2-}$ [3,4,5]. However, due to the lack of key parameters of these species, the nature of the early age phase transformations can mainly be glimpsed by simulations [4,6]. So, to validate the findings in simulations and expand our understanding, these parameters are urgently needed. Electrical conductivity, indicating the compositions of the solution, is an easily acquired property to decouple these phase transformations processes. An accurate interpretation of electrical conductivity relies on predetermined equivalent conductivity of each ion in the solution. However, the ionic conductivities of two aqueous silicate species, ${\left[\mathrm{SiO}{\left(\mathrm{OH}\right)}_{3}\right]}^{-}$ and ${\left[\mathrm{Si}O_{2}{\left(\mathrm{OH}\right)}_{2}\right]}^{2-}$, have been rarely discussed.

Quantitively, the equivalent conductivities of these two silicate species can be measured via two approaches [7,8,9,10]. On the one hand, in a sufficiently diluted system (i.e., for ideal behavior), according to Kohlrausch’s law of the independent migration of ions, the overall conductivity (κ, in $\mathrm{mS}\cdot cm^{-1}$) can be described by the sum of the individual contributions of all present ions. Based on this law, $\lambda_{i}$, the equivalent single-ion conductivity at infinite dilution, is usually calculated from the conductivities of sodium silicate aqueous solutions. But it should be noted that the accuracy in the measurements is contingent upon adherence to four crucial prerequisites as follows: (1) The meticulous determination of the exact compositions of electrolyte solutions through thermodynamic computations; (2) high precision in the determination of electrical conductivity; (3) stability of experimental conditions, including controlled temperature, mitigation of dissolved $CO_{2}$ concentration in the solution, and rigorous control of electrical potentials during the sampling process; (4) the accuracy of the chosen conductivity model for mixed electrolyte solutions. In [8], Zaytsev et al. have compiled the conductivities of sodium silicate aqueous solutions of different studies at 291.15 K, which were correlated to the mass percentage of sodium silicate solid. However, their data does not lead to the equivalent conductivities of the different silicate anions, as the crystallized water content in the silicate solid was not specified. Harman [9] also measured the conductivity of sodium silicate aqueous solution with varying $Na_{2}O:\mathrm{Si}O_{2}$ ratios at 298.15 K. But the polymerization of monomeric silicate and interconversion between different silicate anions were not introduced into the calculation for the mobility of silicate ions. With Kohlrausch’s law of the independent migration of ions and thermodynamic data for silicate [11], Harman’s data (see Table I of [9]) for the low concentration of ratio 1:1 sodium silicate solution ($N_{w}$ = 0.005 and 0.01, where $N_{w}$ indicates the content of 1000 g of water of gram-equivalent Na) suggests that the equivalent conductivity of ${\left[\mathrm{SiO}{\left(\mathrm{OH}\right)}_{3}\right]}^{-}$ is about 19.594 $S\;cm^{2}\;\mathrm{mo}l^{-1}$. However, it should be noted that their measured conductivities of NaOH aqueous solution (see Table II in [9]) are lower than current widely accepted values, e.g., the data given in [12]. Similar experiments have been reproduced by Ukihashi [10] and Lian et al. [7], but the concentrations in the latter are too high to achieve $\lambda_{i}$.

On the other hand, from the thermodynamic point of view, the Nernst–Einstein equation, Equation (S-1), relates the self-diffusion coefficient of the ions, $D_{i}$ (in $m^{2}\;s^{-1}$), with $\lambda_{i}$ without considering the electrical neutrals. Put differently, in an electrolyte solution, $D_{i}$ and $\lambda_{i}$ serve as two distinct parameters that essentially describe the same migration ability of ion i in an external electrical field. For most ions, measurement of $\lambda_{i}$ provide accurate values for $D_{i}$ [13]. $D_{i}$ of silicate ions is crucial for diagenetic modeling [14,15], estimating diffusive fluxes of dissolved chemical species (e.g., in alkali–silica reaction [16] or the self-healing of cementitious materials [17]), analyzing cement hydration at early age [18,19,20,21], and investigating the processes of phase transformations of silicates (e.g., calcium silicate hydrates (C-S-H) [22]). Table S.1 compares $D_{i}$ of specific silicate species in aqueous solution in related published research and measured value of $\lambda_{{\left[\mathrm{Si}O_{2}{\left(\mathrm{OH}\right)}_{2}\right]}^{2-}}$. It is shown that despite the importance of these parameters in different disciplines, the deviations between the published values of $D_{i}$ are over two orders of magnitude. Moreover, the uncertainty in the measured $D_{i}$ or $\lambda_{i}$ for specific silicate species has not been systematically addressed.

Mills et al. [23] discussed eight methods of measuring $D_{i}$. However, compared with these methods, using the conductivity measurements to derive $\lambda_{i}$ is more convenient as it does not require radioisotopes or confinement for the diffusion flux. Aiming to evaluate $\lambda_{{\left[\mathrm{Si}O_{2}{\left(\mathrm{OH}\right)}_{2}\right]}^{2-}}$ and $\lambda_{{\left[\mathrm{SiO}{\left(\mathrm{OH}\right)}_{3}\right]}^{-}}$, a conductivity model for mixed electrolytes is primarily proposed and then validated with NaOH solutions and $\mathrm{NaOH}\text{-}{\mathrm{Na}}_{2}CO_{3}$ solutions. Considering the errors stemming from the aforementioned four scenarios, $\lambda_{{\left[\mathrm{Si}O_{2}{\left(\mathrm{OH}\right)}_{2}\right]}^{2-}}$ and $\lambda_{{\left[\mathrm{SiO}{\left(\mathrm{OH}\right)}_{3}\right]}^{-}}$ are further calculated via the conductivities of $\mathrm{NaOH}\text{-}Na_{2}\mathrm{Si}O_{4}\text{-}H_{2}O$ mixtures at different temperatures. The advantages and limitations of calculations and measurements are addressed subsequently. These analyses filled the gap in the quantitative investigation of thermophysical and thermochemical properties of silicate materials.

## 2. Materials and Methods

### 2.1. Materials

In the periodic table of elements, carbon and silicon belong to the same main group. So, their corresponding anions share a similar structure and electrically conductive properties. Also, the equivalent conductivities of $CO_{3}^{2-}$ and $\mathrm{HC}O_{3}^{2-}$ have been frequently investigated. Therefore, sodium carbonate solutions with different pH values are ideal references to validate the conductivity model for mixed electrolytes. By dissolving into different solutions with designed pH values, sodium metasilicate pentahydrate could offer varying concentrations of monomeric silicate species. Sodium metasilicate pentahydrate is the most frequently used silicon-containing chemical in investigations of phase transformations of silicates in aqueous solutions. In addition, sodium hydroxide solutions and potassium chloride solutions are the most used chemicals to adjust pH value and calibrate electrical conductivity, respectively. In all experiments, four groups of electrolyte solutions were prepared. The corresponding raw substances and the compositions of solutions are shown in Table 1 and Table 2, respectively.

For calibration, KCl solutions (group K1 to K3) with varying concentration were acquired by dosing different volumes of 3.31 mol/kg KCl aqueous solution into treated water, following the procedures below. A total of 50 mL of mother KCl solution was first placed in a 100 mL high density polyethylene container, connecting a homemade gas-washing bottle with PTFE tube. Nitrogen (purchased from Air Liquid (Liège, Belgium), with purification of 99.8%), was bubbled through the two bottles for 1 h to remove dissolved $CO_{2}$. The container was then connected to an automatic burette (ABU80, produced by Radiometer Copenhagen (Copenhagen, Denmark) with a resolution of 0.1 μL) with a PTFE tube. The concentration of KCl solutions is designed to match the conductivities of groups S1 to S7 at different temperatures.

NaOH solutions (group N1 to N7) are prepared in a similar fashion. But to prevent carbonation during dosing, the bottle of NaOH solution was successively connected to an empty 500 mL bottle and two gas-washing bottles. The concentrations of $OH^{-}$ were designed by setting pH values in the range of 12 to 13, as the majority of experimental investigations on phase transformation of C-S-H were conducted in this pH range [22,31,32].

Sodium carbonate and sodium silicate solutions are prepared by dissolving solids in water under nitrogen atmosphere. $Na_{2}CO_{3}$ and $Na_{2}\mathrm{Si}O_{3}\cdot 5H_{2}O$ solids were used as received. Both of their initial concentrations were designed to be below 5 mmol/kg. During the measurements of group S0, sodium silicate solids were sequentially added into the reactor. The amounts of solids were designed to minimize the impact of polymerization. Sodium carbonate solids were added to sodium hydroxide solutions at designed mix ratios to verify the conductivity model for mixed aqueous electrolytes.

A total of 250 mL demineralized water (conductivity < 0.5 $\mu S\;cm^{-1}$), treated by the apparatus produced by EuroWater Belgium N.V. (Nazareth-Eke, Belgium), was further boiled for 10 min, and distilled to remove as much dissolved $CO_{2}$ as possible. A 100 mL GL45 bottle was used for collecting distilled water and was sequentially connected to an empty 250 mL GL45 bottle and a gas-washing bottle. The collecting bottle was filled with $N_{2}$ before distillation. The distilled water was also bubbled with $N_{2}$. A 30 mL syringe was used to transfer the processed water to the reactor, which had been flushed with $N_{2}$ for 10 min. The quantity of dosed water was shown by the difference in weight of this syringe with and without water (measured immediately after dosing).

### 2.2. Conductivity Measurements

A closed jacketed 100 mL glass reactor with a PTFE liner was used to avoid carbonation, as shown in Figure S.1. The measured solution was swept by $N_{2}$. All the containers in the current experiments, except the gas-washing bottles, were tightly sealed. All solutions were mildly stirred magnetically during the measurements. The temperatures in all measurements were designed to the range of 278.15 to 308.15 K. The temperatures of the solution were kept constant via water, circulating through a heating unit (Omron E5CSV, OMRON Europe B.V., Hoofddorp, The Netherlands) and a cooling box (Mobicool ME24, Mobicool, Emsdetten, Germany). The fluctuation of the temperature was limited below 0.1 K. The real temperature of the measured solution was read by a Pt100 sensor, connected to a magnetic stir (VWR VMS-C7, Avantor, Radnor, PA, USA). The real measuring temperatures might slightly drift from the designed values due to heat loss in circulating water and temperature overcompensation.

A two-polar conductivity cell (SK10B, with the cell constant $C=1\;cm^{-1}\;\pm \;30\%,$ according to the manufacture) was bought from Consort. Before each test, it was conditioned in 0.1 mol/L HCl for 1 h and then rinsed with deionized water. The cell was connected to a BNC female connector (RS components, Brussels, Belgium). The working sensor and working electrode were connected to the shield of the BNC connector, while the lead of counter electrode and the reference electrode were attached to the inner pin [33]. The rest of the electrodes were isolated in a grounded Faraday cage.

The resistances of the solutions were read from the peaks or the intersection points of Nyquist plots. The spectrum was measured via a Gamry Interface 1000 potentiostat (Gamry Instruments Inc., Warminster, PA, USA) (1 MHz to 1 Hz, 10 points per decade, AC Voltage of 10 mV r.m.s.). For each composition of analyte, the spectrum was repeatedly plotted four times once the temperature of solution reached the designed value and there were no visible undissolved particles.

### 2.3. Data Processing

The activity of aqueous species were calculated by the extended Debye–Hückel equation (Helgeson), as shown in Equations (S-2)–(S-5) [29]. Tables S.2 and S.3 present the corresponding parameters for activity coefficient calculation. GEM-Selektor v.3 (GEMS3) with databases of PSI/Nagra (TDB2020) and cemdata (18.01) [11] was used to calculate solution compositions. The corresponding thermodynamic data for calculation is presented in Table S.4. The regression analyses were conducted via the least-square method solver ‘lsqlin’ in MATLAB (23.2.0.2515942 (R2023b)) with restricting conditions. A main indicator for evaluating regression is numerical deviation. It was defined as the absolute value of difference between the measured conductivity and the calculated conductivity.

## 3. Conductivity Models

A conductivity model is indispensable for ionic equivalent conductivity measurement, as it correlates the composition of electrolyte and the corresponding electrical conductivity. Up to now, a large number of models have been proposed to interpret the conductivity of electrolyte solutions [34,35,36]. The simplest one is kohlrausch’s law of the independent migration of ions, as shown in Equation (1):

(1) $$\kappa =\sum_{i}c_{i}\cdot z_{i}\cdot \lambda_{i}$$

where $z_{i}$ is the valency of ion i, $c_{i}$ is the molarity (expressed in mol/L) of the ion and $\lambda_{i}$ (expressed in $S\;cm^{2}\;\mathrm{mo}l^{\text{-}1}$) is the equivalent single-ion conductivity at infinite dilution [37,38]. It says that the ions will not influence each other’s movement in a sufficiently diluted system (i.e., for ideal behavior). In a more concentrated solution, it overestimates the conductivity. But even so, in [14] the calculated conductivity of a sodium carbonate solution (10 mmol/L) still matches the trend of measured values using Equation (1). At low concentrations, Kohlrausch’s law describes the molar conductivities ($\Lambda_{m}$, in $\mathrm{mS}\;cm^{-1}\;\mathrm{mo}l^{-1}$) of strong electrolytes:

(2) $$\Lambda_{m}=\Lambda_{m}^{0}-Kc^{\frac{1}{2}}$$

where $\Lambda_{m}^{0}$ is the limiting molar conductivity and K is Kohlrausch constant (expressed in $S\;cm^{2}\;\mathrm{mo}l^{-1.5}$) [38]. This expression is identical to the Debye–Hückel–Onsager equation, where K is replaced with $A+B\Lambda_{m}^{0}$ (A and B are parameters determined by temperature and properties of solvent, expressed in $S\;cm^{2}\;\mathrm{mo}l^{\text{-}1.5}\;L^{0.5}$ and $\mathrm{mo}l^{-0.5}\;L^{0.5}$, respectively [39]). Equation (2) is reliable for c < 1 mmol/L [40]. For higher concentrations, the short-range interactions between a central ion and its surrounding ionic atmosphere have to be considered (see relaxation and electrophoretic effects in [34]). When c > 1 mol/L, Robinson and Stokes’ conductivity model (abbreviated as RS model, shown in the following Equations (3)–(6) gives good agreement with experimental results with proper correction of viscosity [41,42,43]:

(3) $$\Lambda_{m}=\Lambda_{m}^{0}-\frac{\left(B_{1}\Lambda^{0\;}+B_{2}\right)c^{0.5}}{1+Båc^{0.5}}$$

(4) $$B_{1}=\frac{8.20\;\times \;10^{5}}{{\left(\varepsilon T\right)}^{1.5}}$$

(5) $$B_{2}=\frac{82.5}{\eta {\left(\varepsilon T\right)}^{0.5}}$$

(6) $$B=\frac{50.29}{{\left(\varepsilon T\right)}^{0.5}}$$

where ε is the dielectric constant (permittivity) at zero frequency, η is the viscosity of water (expressed in mPa s equal to centipoise) and å is the distance of closest approach of the ions, expressed in angstroms (3.5 Å for NaOH). å is a constant for each solute at all temperatures from 278.15 K to 338.15 K. It is not always practical to record viscosity in parallel with conductivity measurement, which limits the application of RS models for highly concentrated solutions.

For mixed electrolyte solutions modeling of the conductivities is much more complicated [44]. The computation of the numerator of the relaxation effect is mathematically the most difficult part of electrolyte theory [34]. Therefore, many fitting parameters (e.g., the frictional parameter [45]) have been introduced to the derivation [46,47]. McCleskey et al. [48] evaluated the performances of 11 conductivity models with a wide range of natural water. They found that the model they proposed in 2011 [49] was applicable to the widest pH and temperature range as well as the highest ionic strength (up to 1 mol/L). For calculating the conductivity of a mixed electrolyte solution, they replaced $c_{i}$ in Equation (1) with $m_{i}$ (speciated molality of the i ion). But this procedure introduces anomalous units in κ.

Sodium silicate solution is a mixed electrolyte solution, due to the hydrolysis of silicate ions. To model its conductivity, two terms are needed. One is for quantifying the conductivity contribution of the background NaOH solution. Another term should be built for calculating the conductivity of other ions. In the investigation of phase transformation of silicate, e.g., C-S-H, the pH value of solutions is always set above 12.0 to prevent silicate polymerization, while the concentration of silicate ions is frequently defined below 10 mmol/L [22,31,50,51]. In such systems, especially under elevated temperatures, the concentrations of silicate ions are always much lower than that of hydroxide ions. Thus, it is assumed that the relaxation and electrophoretic effects introduced by cations and silicate ions are negligible. In other words, the conductivity property of these mixed electrolyte solutions could be considered as the superposition of concentrated NaOH solution and diluted silicate solutions, where only the non-ideal conductivity (with respect to Equation (1)) of NaOH solution should be calculated. So, the key part of such a conductivity model must precisely describe the conductivity of NaOH solution with different concentrations under varying temperatures. Simply speaking, this conductivity model should be expressed as Equation (11), where the molar conductivity of NaOH solution ($\Lambda_{m}$) can be obtained from the McCleskey’s conductivity model (Equations (7)–(10), where m stands for the apparent molality, t indicates the temperature in °C [49]), and $\Lambda_{\mathrm{calibrated}}$ indicates the calibrated McCleskey’s molar conductivity NaOH at a specific temperature.

(7) $$\Lambda_{m}^{0}\left(t\right)=0.006936t^{2}+3.872t+148.3$$

(8) $$A=0.01018t^{2}+0.67421t+56.76$$

(9) B=0.2

(10) $$\Lambda_{m}={\;\Lambda }_{m}^{0}\left(t\right)-A\frac{m^{1/2}}{1+Bm^{1/2}}$$

(11) $$\kappa =\sum_{i}c_{i}\cdot z_{i}\cdot \lambda_{i}+{\;c}_{\mathrm{NaOH}}\cdot \Lambda_{\mathrm{calibrated}}(\Lambda_{m})$$

## 4. Results and Discussion

### 4.1. Validation of the Conductivity Model for NaOH Solution

First of all, the cell constant, C, was obtained by calibrating the conductivity electrode with KCl solution at different temperatures. The compositions of the solutions were presented in Table S.5. According to the calibration curves in Figures S.4–S.7, $C=1.055\;cm^{-1}$ gives acceptable agreement with experiments at 277.65 K and 293.55 K, and 308.45 K. The goodness of fit was evaluated by coefficient of determination, $R^{2}$, as shown in Equation (S-8). For these three temperatures, the value of $R^{2}$ is 0.9998, 0.9999, and 0.9972, respectively.

**Figure 1:**
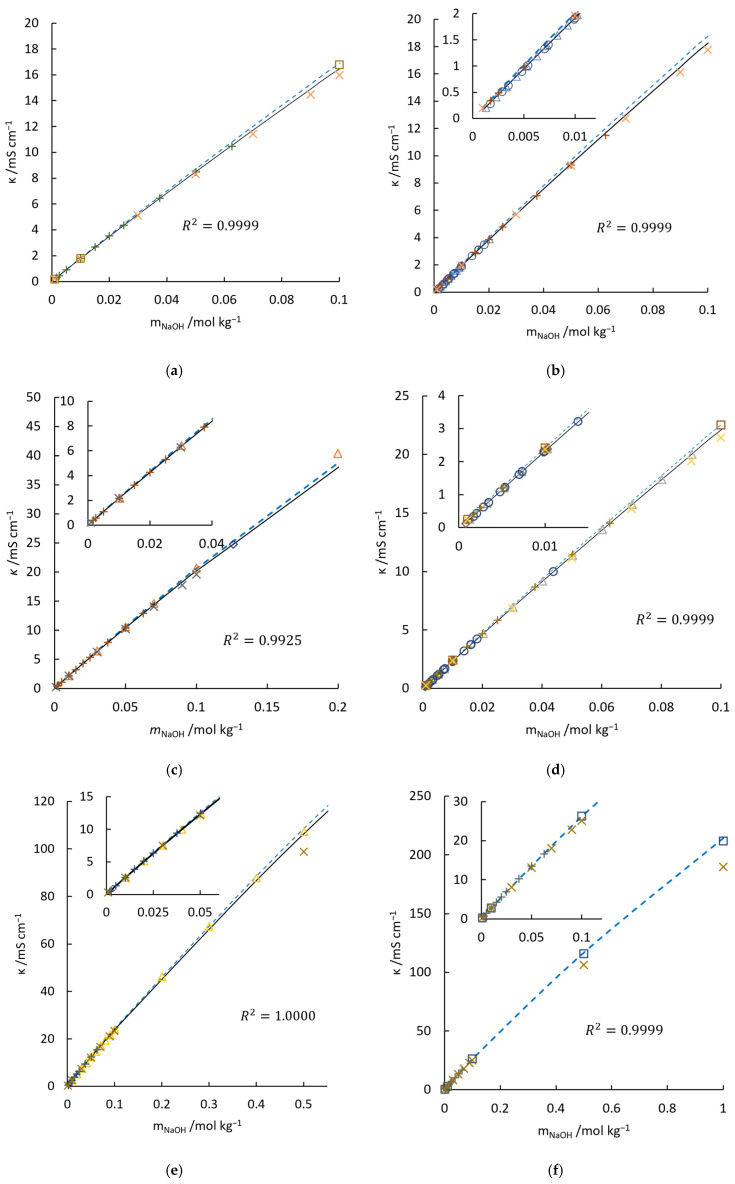
Comparisons of conductivities of NaOH aqueous solution from published data, McCleskey’s model, calibrated McCleskey’s model, and RS model at 283.15 K (**a**), 288.15 K (**b**), 293.15 K (**c**), 298.15 K (**d**), 303.15 K (**e**), 308.15 K (**f**). □, McCleskey’s measurements [49]; +, measurements from [8]; Δ, measurements from [52]; ○, Stokes’ measurements [53]; x, calculated conductivity from RS model [34]. The data referred to by these symbols under different temperatures are distracted by different colors. In addition, ----, McCleskey’s model [49]; —, calibrated McCleskey’s model.

Thereafter, to verify Equation (11), published conductivities of NaOH aqueous solutions of different temperatures [3,17,37,43,44], are compared with the original McCleskey’s model (blue dashed line), calibrated model (black line), and RS model (orange cross) in Figure 1. As shown in Figure 1a,d, McCleskey’s measured values (empty squares) are obviously higher than the ones from other works at 283.15 K and 298.15 K, respectively. One of the main reasons could be attributed to the overrepresented value measured at the temperatures concerned. That is why this model needs to be calibrated.

The correction coefficients and the coefficient of determination, $R^{2}$, of each temperature are listed in Table 3. At 308.15 K, as shown by Figure 1f, McCleskey’s model fits experimental results very well. Thus, no further correction is needed at this temperature. The conductivities of NaOH aqueous solution at 278.15 K are rare. The respective correction coefficient is acquired by taking the new measured values from current work at 277.65 K as reference. For other temperatures, the correction can be approximated by a central differencing scheme. It can be seen from the dark line, such as the one in Figure 1, that after calibration, the model agrees with other published data very well.

It should be noted that the formation of the electroneutrality ion pair NaOH@ (here the nomenclature in GEMS3 is introduced, where ‘@’ stands for electrical neutrals [11]) should not be neglected. For instance, adding 0.5 mol NaOH solid into 1 kg water under 278.15 K, the amount of NaOH@ takes 11.87% of the total amount of Na-containing species, with the thermodynamical data given in [11]. Thus, the apparent molality (or molarity) should include the amount of disassociated NaOH and NaOH@. As McCleskey’s model deduced from the fitting of measured conductivities of solutions of 0.001, 0.01, 0.1, 0.5, and 1 mol/kg, ‘m’ in Equation (10) should stand for the apparent molality. Also, ‘c’ in Equation (11) must refer to the apparent molarity. On the contrary, the Robinson–Stokes’ model is derived from the interaction of particles. Thus, this model can only be used in fully ionized electrolytes with real concentration [42].

Theoretically, the RS model should predict the exact conductivity at diluted concentrations. But in the inset of Figure 1b, its results are higher than the average of experimental values. Since this deviation is unnoticeable under other temperatures, it can be inferred that the overestimated conductivity at low concentrations should not be attributed to the inaccurate ‘å’. In addition, at low concentration, viscosity correction can be ignored. On extending the calculation to higher concentrations, conductivities predicted by the RS model are lower than measured values. Although a viscosity correction can compensate this offset [54], the formation of ion-pair has already placed the theory on shaky ground. On the contrary, a fitted model, e.g., McCleskey’s model, covers all sophisticated calculations with fitted parameters. Therefore, based on the discussion above, the second term in Equation (11) applies to predicting the conductivities of NaOH solution with indicated concentration at 278.15 K to 308.15 K.

After being calibrated with published data, McCleskey’s conductivity model for NaOH solution is verified with new measurements in the current work, as shown in Figure S.9. The compositions of the solutions and uncertainties are shown in Table S.7. These new measurements prove the applicability of the calibrated model and conductivity measuring method.

### 4.2. Validation of the Conductivity Model with $\mathrm{NaOH}\text{-}Na_{2}CO_{3}\text{-}H_{2}O$ Electrolytes

**Figure 2:**
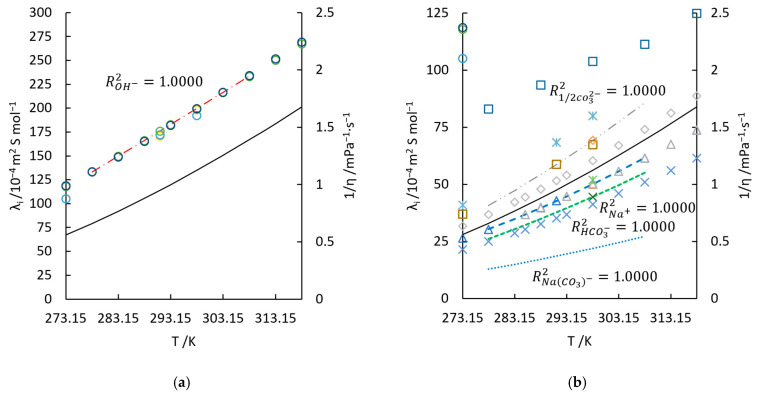
Equivalent conductivities of ions, $\lambda_{i}$, and reciprocal of water viscosity, 1/η, at temperature range of 298.15 K to 318.15 K. In (**a**): ○, $\lambda_{OH^{-}}$ [34]; ○, $\lambda_{OH^{-}}$ [56]; ○, $\lambda_{OH^{-}}$ [57]; **○**, $\lambda_{OH^{-}}$ [58]; ·ꟷ·ꟷ, Equation (14); ꟷ, 1/η [34]. In (**b**): Δ, $\lambda_{Na^{+}}$ [34]; Δ, $\lambda_{Na^{+}}$ [12]; Δ, $\lambda_{Na^{+}}$ [59]; **- - - **, Equation (13); □, $\lambda_{\mathrm{Cl}O_{4}^{-}}$ [34]; □, $\lambda_{\mathrm{Cl}O_{4}^{-}}$ calculated from $D_{\mathrm{Cl}O_{4}^{-}}$ [23]; □, $\lambda_{\mathrm{Cl}O_{4}^{-}}$ [60]; ×, $\lambda_{\mathrm{HC}O_{3}^{-}}$ [12]; ×, $\lambda_{\mathrm{HC}O_{3}^{-}}$ [13]; ---, $\lambda_{\mathrm{HC}O_{3}^{-}}$ Equation (16); ◊, $\lambda_{1/2\;CO_{3}^{2\text{-}}}$ [12]; ◊, $\lambda_{1/2\;CO_{3}^{2\text{-}}}$ [13]; ··ꟷ··ꟷ, Equation (15); ······, Equation (17); 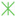, $\lambda_{\mathrm{HS}O_{4}^{-}}$ [12]; 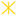, $\lambda_{1/2\;SO_{4}^{2\text{-}}}$ [34]; 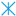, $\lambda_{1/2\;SO_{4}^{2\text{-}}}$ [23]; ꟷ, 1/η [34].

To verify Equation (11) for mixed solutions, the conductivity of NaOH and $Na_{2}CO_{3}$ mixtures are measured at different temperatures. Carbon and silicon share analogous physico-chemical properties as indicated by their position in the periodic table of elements. Therefore $Na_{2}CO_{3}$ solution was used for the following verification. Also, $\lambda_{1/2\;CO_{3}^{2-}}$ and $\lambda_{\mathrm{HC}O_{3}^{-}}$ at 298.15 K have been widely investigated, although the data at other temperatures has been, again, rarely published. Zeebe et al. [13] found that these data can be approximated from the viscosity of water based on the Stokes–Einstein equation:

(12) $$D_{i}=\frac{k_{B}T}{6\pi \eta r}$$

where $k_{B}$ is Boltzmann’s constant, η is the shear viscosity of water and r is the radius of the spherical radius of the ion [13] (see Figures 9 and 10 in [13]). But Zeebe’s work underestimated $\lambda_{1/2\;CO_{3}^{2-}}$ and $\lambda_{\mathrm{HC}O_{3}^{-}}$, comparing with the experimental values in [34,40] as shown in Figure 2. The equivalent conductivities of carbonate species are thus estimated via η in the following calculations. Other than $CO_{3}^{2-}$ and $\mathrm{HC}O_{3}^{-}$, $\mathrm{Na}{\left(CO_{3}\right)}^{-}$ also contribute to the bulk conductivity. Its equivalent conductivity is 22.0 $S\cdot cm^{2}\cdot \mathrm{mo}l^{-1}$ at 298.15 K [55]. Its values at other temperatures are also estimated in the same way. Although Equation (12) suggests that the equivalent conductivity of heavier ions, (compared to $OH^{-}$), tends to be highly related to the viscosity of water, $\lambda_{i}$ calculated solely from η cannot always stand for the correct conductance properties. For instance, the equivalent conductivity of $SO_{4}^{2-}$, whose trend of temperature dependence has already differed away from the curve of 1/η, as shown in Figure 2b. Nonetheless, Equation (12) offers good initial values for estimation of accurate $\lambda_{i}$. The dashed curves in Figure 2 represent the temperature dependence of equivalent conductivity of the concerned ion. The fitted $\lambda_{i}$ for $Na^{+}$, $OH^{-}$, $1/2\;CO_{3}^{2-}$, $\mathrm{HC}O_{3}^{-}$, and $\mathrm{Na}{\left(CO_{3}\right)}^{-}$ are:

(13) $$\lambda_{Na^{+}}=-4\;\times \;10^{-5}t^{3}+0.0082t^{2\;}+0.7678t+26.441,\;\left(R^{2}=1.0000\right).$$

(14) $$\lambda_{OH^{-}}=0.0028t^{2}+3.2344t+117,\;\left(R^{2}=1.0000\right).$$

(15) $$\lambda_{1/2\;CO_{3}^{2-}}=0.0072t^{2\;}+1.2099t+34.523,\;\left(R^{2}=1.0000\right).$$

(16) $$\lambda_{\mathrm{HC}O_{3}^{-}}=0.0047t^{2}+0.777t+22.168,\;\left(R^{2}=1.0000\right).$$

(17) $$\lambda_{\mathrm{Na}{\left(CO_{3}\right)}^{-}}=-9\;\times \;10^{-6}t^{3}+0.003t^{2}+0.3699t+11.034,\;\left(R^{2}=1.0000\right).$$

**Figure 3:**
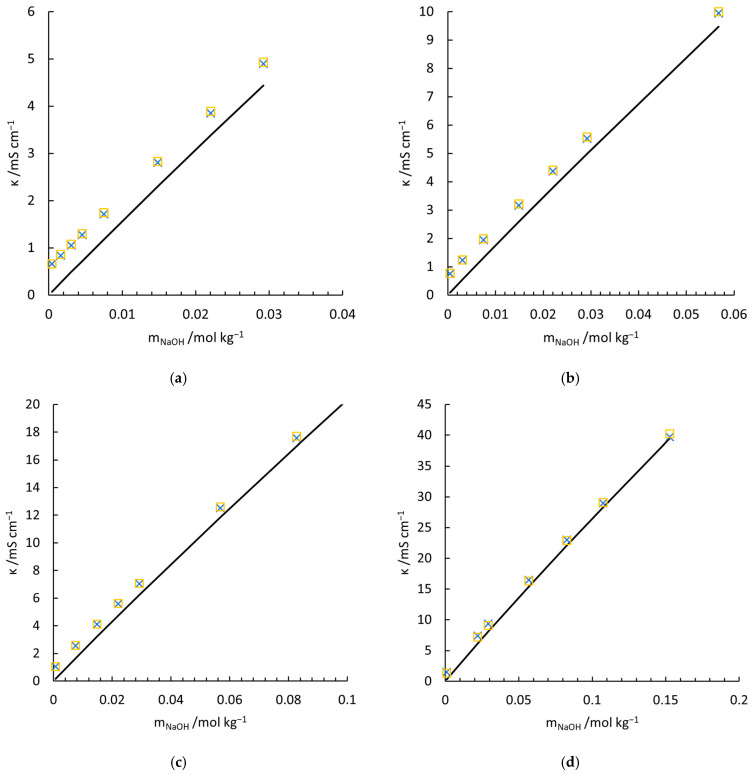
Measured and calculated conductivity of $\mathrm{NaOH}\text{-}Na_{2}CO_{3}\text{-}H_{2}O$ electrolytes at 277.15 K (**a**), 282.65 K (**b**), 293.55 K (**c**), and 308.45 K (**d**). □, Equation (11); ×, measured conductivities in the current work; —, conductivity contribution of NaOH solution with calibrated McCleskey’s model.

Figure 3 illustrates the measured conductivity of $\mathrm{NaOH}\text{-}Na_{2}CO_{3}\text{-}H_{2}O$ solutions (indicated by crosses), conductivity contribution of NaOH calculated by calibrated McCleskey’s model (dark solid line) and the conductivity predicted by Equation (11) (yellow square). The calculated conductivities match the measured value very well. The compositions and corresponding uncertainties are shown in Tables S.8 and S.9, respectively.

### 4.3. Calculation of $\lambda_{{\left[\mathrm{SiO}{\left(\mathrm{OH}\right)}_{3}\right]}^{-}}$ and $\lambda_{{1/2\;\left[\mathrm{Si}O_{2}{\left(\mathrm{OH}\right)}_{2}\right]}^{2\text{-}}}$ from Conductivities of $\mathrm{NaOH}\text{-}Na_{2}\mathrm{Si}O_{3}\text{-}H_{2}O$ Solutions

The accuracy of calculated $\lambda_{{\left[\mathrm{SiO}{\left(\mathrm{OH}\right)}_{3}\right]}^{-}}$ or $\lambda_{{1/2\;\left[\mathrm{Si}O_{2}{\left(\mathrm{OH}\right)}_{2}\right]}^{2\text{-}}}$ relies on proper estimated initial values as input for iterations. Their magnitude can be estimated from $D_{i}$, which is often approximated using the Stokes–Einstein relation [13]. Also, their values can be assessed from the equivalent conductivity of anions with similar atomic structures. According to the location of silicon in the periodic table (Table 4), the equivalent conductivity of ${\left[\mathrm{SiO}{\left(\mathrm{OH}\right)}_{3}\right]}^{-}$ should be lower than that of ${\left[{\mathrm{PO}}_{2}{\left(\mathrm{OH}\right)}_{2}\right]}^{-}$ (36.00 $S\;cm^{2}\;\mathrm{mo}l^{-1}$), as the silicon–oxygen bond is longer than the phosphorus–oxygen bond; thus, $r_{{\left[\mathrm{SiO}{\left(\mathrm{OH}\right)}_{3}\right]}^{-}}$ would be larger than $r_{{\left[{\mathrm{PO}}_{2}{\left(\mathrm{OH}\right)}_{2}\right]}^{-}}$. Furthermore, as all the single charged anions of Si, P, S, and Cl are tetrahedral arranged with 4 oxygen atoms, their equivalent conductivities should successively increase as the sizes decrease. Furthermore, in the third period from group 14 to 17 in the periodic table (from Si to Cl), for each additional electron, the equivalent conductivity of the monovalent anion for this element increases by 15.65 $S\;cm^{2}\;\mathrm{mo}l^{-1}$. Exclusively based on this trend, it can be estimated that $\lambda_{{\left[\mathrm{SiO}{\left(\mathrm{OH}\right)}_{3}\right]}^{-}}\;\approx \;20.47\;S\;cm^{2}\;\mathrm{mo}l^{-1}$. In addition, by disassociating another proton from hydroxyl groups, the conductivities of anions marked an increase. For instance, at 273.15 K the conductivity of ${\left[CO_{3}\right]}^{2-}$ is 3.11 times higher than that of ${\left[CO_{2}\left(\mathrm{OH}\right)\right]}^{-}$ (for ${\left[{\mathrm{PO}}_{3}\left(\mathrm{OH}\right)\right]}^{2-}$ and ${\left[{\mathrm{PO}}_{2}{\left(\mathrm{OH}\right)}_{2}\right]}^{-}$ the ratio of their conductivity is 3.17, for ${\left[SO_{4}\right]}^{2-}$ and ${\left[{\mathrm{SO}}_{3}\left(\mathrm{OH}\right)\right]}^{-}$ it is 3.07). So, the conductivity of ${\left[\mathrm{Si}O_{2}{\left(\mathrm{OH}\right)}_{2}\right]}^{2-}$ must also be higher than that of ${\left[\mathrm{SiO}{\left(\mathrm{OH}\right)}_{3}\right]}^{-}$. Thus $\lambda_{{1/2\;\left[\mathrm{Si}O_{2}{\left(\mathrm{OH}\right)}_{2}\right]}^{2-}\;}$ can be estimated about 32.75 $S\;cm^{2}\;\mathrm{mo}l^{-1}$ with the ratio of 3.20.

The value of $\lambda_{{\left[\mathrm{SiO}{\left(\mathrm{OH}\right)}_{3}\right]}^{-}}$ is 35.00 $S\;cm^{2}\;\mathrm{mo}l^{-1}$ at 273.15 K from Greenberg’s calculation [61]. This value is higher than the above estimated value ($\lambda_{{\left[\mathrm{SiO}{\left(\mathrm{OH}\right)}_{3}\right]}^{-}}\;\approx \;20.47\;S\;cm^{2}\;\mathrm{mo}l^{-1}$). In thermodynamical simulations of cementitious materials [16,17], the diffusion of ${\left[\mathrm{Si}O_{2}{\left(\mathrm{OH}\right)}_{2}\right]}^{2-}$ at 273.15 K is cited as $0.7\;\times \;10^{-9}\;m^{2}/s$ from [62]. So, $\lambda_{{1/2\;\left[\mathrm{Si}O_{2}{\left(\mathrm{OH}\right)}_{2}\right]}^{2\text{-}}}$ calculated via the Nernst–Einstein equation ($30.72\;S\;cm^{2}\;\mathrm{mo}l^{-1}$) approximates to the estimation above ($\lambda_{{1/2\;\left[\mathrm{Si}O_{2}{\left(\mathrm{OH}\right)}_{2}\right]}^{2-}}=32.747\;S\;cm^{2}\;\mathrm{mo}l^{-1}$).

In the current work, the equivalent conductivities of ${\left[\mathrm{SiO}{\left(\mathrm{OH}\right)}_{3}\right]}^{-}$ and ${\left[\mathrm{Si}O_{2}{\left(\mathrm{OH}\right)}_{2}\right]}^{2-}$ are calculated by solving eight overdetermined linear equation groups (with respect to group S0 to S7) with the least-square method. For instance, as shown in Table S.10, every row indicates the composition of one analyte solution. By introducing the concentrations of each aqueous species into Equation (11) (after conversation from molality into molarity), every row leads to an equation which has two unknown variables, $\lambda_{{\left[\mathrm{SiO}{\left(\mathrm{OH}\right)}_{3}\right]}^{-}}$ and $\lambda_{{1/2\;\left[\mathrm{Si}O_{2}{\left(\mathrm{OH}\right)}_{2}\right]}^{2-}\;}$. At the temperature of 277.85 K (i.e., group S1), eight independent equations can be established. The solver of ‘lsqlin’ is used to search for a solution of the least squares approximation with constrained conditions. Although mathematically two equations are enough to solve for the two variables, practically this method leads to results which are not consistent with the estimated values. Therefore, $\lambda_{{\left[\mathrm{SiO}{\left(\mathrm{OH}\right)}_{3}\right]}^{-}}$ and $\lambda_{{1/2\left[\mathrm{Si}O_{2}{\left(\mathrm{OH}\right)}_{2}\right]}^{2-}}$ are calculated via the overdetermined equations group, which is composed of eight equations. The calculations are dependent on three input parameters: (1) the ratio, R, between $\lambda_{{1/2\;\left[\mathrm{Si}O_{2}{\left(\mathrm{OH}\right)}_{2}\right]}^{2-}}$ and $\lambda_{{\left[\mathrm{SiO}{\left(\mathrm{OH}\right)}_{3}\right]}^{-}}$, (2) the lower and (3) upper boundary value of the corresponding approximation interval.

R for the two silicate species can be estimated from the periodic table of elements, as shown in Table 5. For silicon, R should be approximated to 1.50. To find an accurate R, the equations in group S0 are solved with R ranging from 0.9 to 1.7. The lower boundary conditions are set to 0 and the equivalent conductivity of ${\left[CO_{2}\left(\mathrm{OH}\right)\right]}^{-}$ and ${\left[CO_{3}\right]}^{2-}$ are used as upper boundary conditions. The results are evaluated via mean square variance (abbreviated as MSV) of each iterative step. As shown in Figure S.10, the best approximation is witnessed when R=1.56, which is close to the estimation of 1.50.

**Figure 4:**
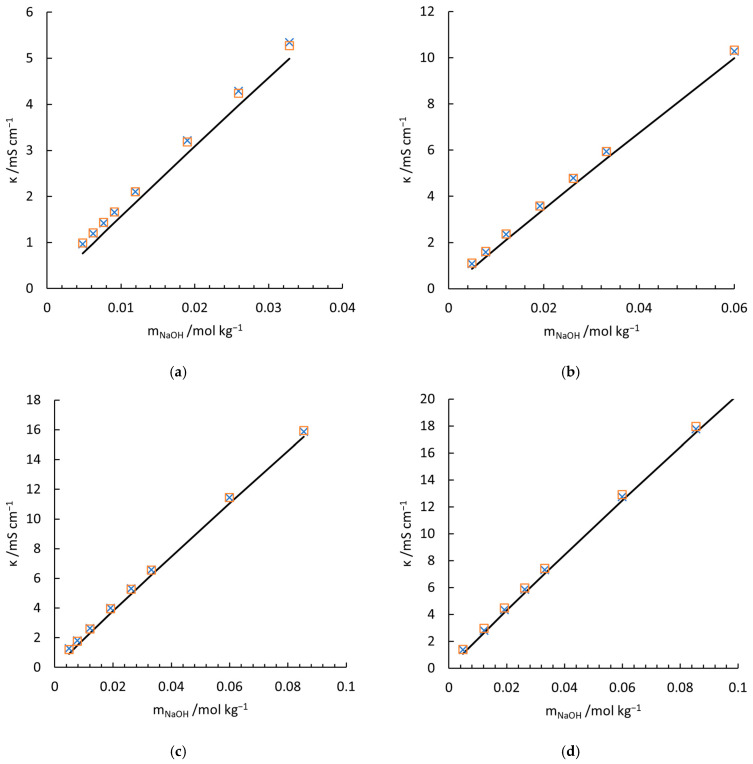

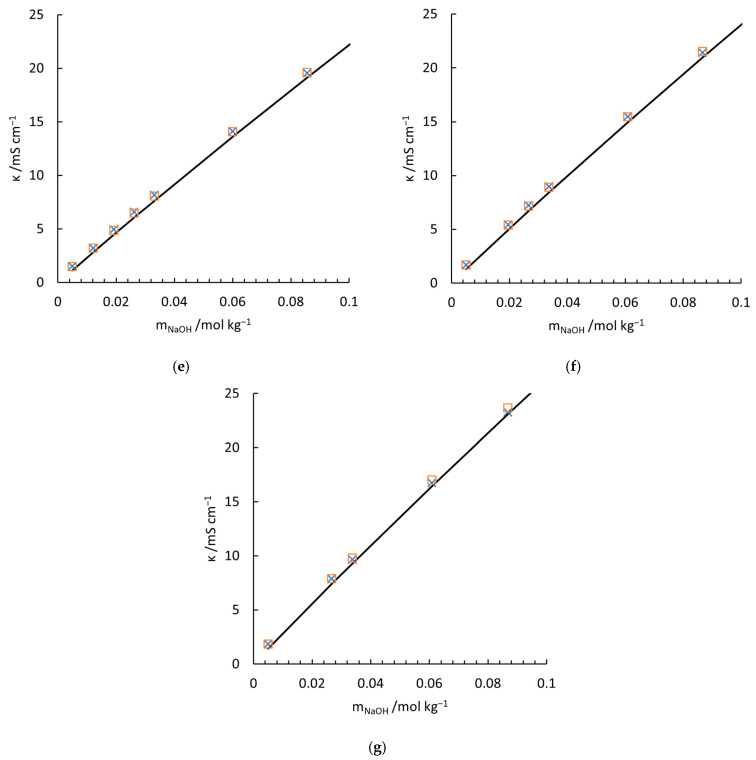
Measured and calculated conductivity of $\mathrm{NaOH}\text{-}Na_{2}\mathrm{Si}O_{3}\text{-}H_{2}O$ electrolytes at 277.85 K (**a**), 282.65 K (**b**), 287.65 K (**c**), 293.65 K (**d**), 298.55 K (**e**), 303.45 K (**f**), and 308.45 K (**g**). □, Equation (12); ×, measured conductivities in the current work; —, conductivity contribution of NaOH solution with calibrated McCleskey’s model.

This R value is then introduced in solving the other seven equations groups (with respect to 7 different measuring temperatures). The grounds for this approach are threefold. Firstly, the Stokes–Einstein relation holds approximately for the temperature dependence of diffusion coefficients of most aqueous ions (e.g., ${\left[CO_{2}\left(\mathrm{OH}\right)\right]}^{-}$ and ${\left[CO_{3}\right]}^{2-}$ [13]). So, η dictates $\lambda_{{1/2\;\left[\mathrm{Si}O_{2}{\left(\mathrm{OH}\right)}_{2}\right]}^{2-}}$ and $\lambda_{{\left[\mathrm{SiO}{\left(\mathrm{OH}\right)}_{3}\right]}^{-}}$ of other temperatures. Secondly, the equivalent conductivities are initially introduced for calculating limiting conductivity electrolytes at infinite dilution. It means the properties of water dominate the conductance of the solution. The boundary conditions for each group are thus approximated based on 1/η. Moreover, as shown in Figure S.11, with an increase in R, the averaged MSV illustrated after the turning point (around 1.5) trends differently from MSV of the maximum deviation for the conductance data of 277.85 K to 303.45 K. It can be inferred that 1.56 is an acceptable value to reconcile these two evaluation parameters. The calculated $\lambda_{{\left[\mathrm{SiO}{\left(\mathrm{OH}\right)}_{3}\right]}^{-}}$ and $\lambda_{{1/2\;\left[\mathrm{Si}O_{2}{\left(\mathrm{OH}\right)}_{2}\right]}^{2-}}$ are shown in Table 5. These data can be fitted to the following two expressions:

(18) $$\lambda_{{\left[\mathrm{SiO}{\left(\mathrm{OH}\right)}_{3}\right]}^{-}}=0.002675t^{2}+0.339061t+10.919579,\;{(R}^{2}=0.9931)$$

(19) $$\lambda_{{1/2\;\left[\mathrm{Si}O_{2}{\left(\mathrm{OH}\right)}_{2}\right]}^{2-}}=0.004180t^{2}+0.529719t+17.059741,\;(R^{2}=0.9931)$$

where t refers to temperature in °C and $R^{2}$ here is used to quantify the goodness-of-fit (with respect to Equation (S-8)), instead of the ratio between the equivalent conductivity of the two species. With these data and the conductivity contribution of NaOH (black solid lines), the measured (indicated by crosses) and predicted value with Equation (11) (empty squares) are compared in Figure 4. In general, Equation (11) with fitted $\lambda_{{\left[\mathrm{SiO}{\left(\mathrm{OH}\right)}_{3}\right]}^{-}}$ and $\lambda_{{1/2\;\left[\mathrm{Si}O_{2}{\left(\mathrm{OH}\right)}_{2}\right]}^{2-}}$ reconciles the experiments well. The largest deviation is witnessed in Figure 4g, which shows the conductivities measured at 308.45 K. One of the main reasons is that to maintain the pH of the solution at a high value (12 to 13), the concentration of NaOH has to be much higher than that at lower temperatures. As discussed above, the theoretical Kohlrausch’s law of the independent migration of ions is no longer applicable for such a high concentration, as well as the superposition principle for the mixture of sodium silicate and sodium hydroxide solutions. The overestimated conductivities are also witnessed in Figure 3d at high concentrations, which supports the statement. In addition, the temperature fluctuations (0.3–0.5 K) in the calibration of McCleskey’s conductivity model for NaOH contributes to the deviation. Although at low concentration the introduced conductivity deviation is neglectable, higher concentrations may amplify the inconsistency. This is why deviations always arise with elevated concentration at every measuring temperature (Figure 4a–g).

**Figure 5:**
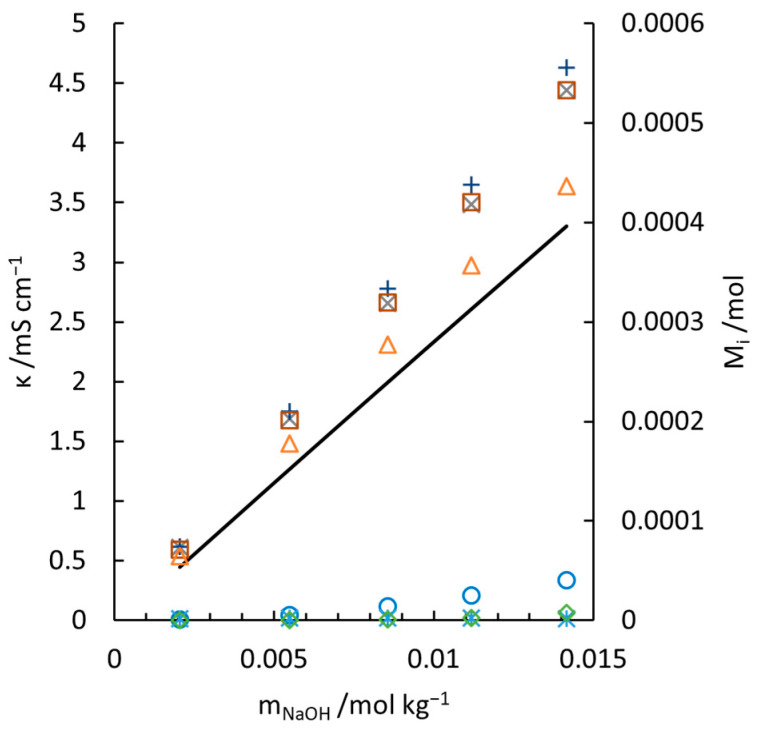
Measured and calculated conductivities of sodium silicate solution, as well as the amounts, $M_{i}$, of four silicate aqueous species with increasing concentration of sodium silicate at 298.55 K. —, conductivity contribution of NaOH solution with calibrated McCleskey’s model. ×, measured conductivities in the current work; +, measured conductivities [61]; □, Equation (11); Δ, amount of ${\left[\mathrm{SiO}{\left(\mathrm{OH}\right)}_{3}\right]}^{-}$; ○, amount of ${\left[\mathrm{Si}O_{2}{\left(\mathrm{OH}\right)}_{2}\right]}^{2-}$; 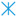 amount of SiO_2_@; ◊, amount of ${\left[Si_{4}O_{10}\right]}^{4-}$.

To compare the results with Ukihashi’s work [10], the conductivities of the $\mathrm{NaOH}\text{-}Na_{2}\mathrm{Si}O_{3}\text{-}H_{2}O$ solutions (group S0) at low concentrations are measured and shown in Figure 5. The amount of each aqueous silicate species is also illustrated. In group S0, the concentrations of silicate species are designed to below the critical level above which oligomers start to form massively. Even with a conservative estimation, where $\lambda_{{\left[\mathrm{SiO}{\left(\mathrm{OH}\right)}_{3}\right]}^{-}}=\lambda_{{1/2\;\left[\mathrm{Si}O_{2}{\left(\mathrm{OH}\right)}_{2}\right]}^{2-}}=35\;S\;cm^{2}\;\mathrm{mo}l^{-1}$, Greenberg’s work (indicated by crosses) overvalues the equivalent conductivity of ${\left[\mathrm{Si}O_{2}{\left(\mathrm{OH}\right)}_{2}\right]}^{2-}$, referring to the left vertical axis. A temperature discrepancy of 0.4 K between his work and this work cannot account for such a high deviation. Our work, on the contrary, perfectly models the experiments and fits the trend of Ukihashi’s work (see Figure 3 in [10]. As there is no tabulated conductivity data presented, accurate data comparison is not executable).

## 5. Error Analysis

### 5.1. The Regression Analysis

In the current work, the maximum deviation is selected as the most representative indicator to estimate the quality of regression. The solver of ‘lsqlin’ is used to search for solutions of the least squares approximation with constrained conditions. In theory, the deviation between calculation and experiments should be normalized to the unit concentration of silicates. However, although the concentration of each silicate species is easily available with thermodynamical calculation practically, the relative concentration of ${\left[\mathrm{SiO}{\left(\mathrm{OH}\right)}_{3}\right]}^{-}$ to ${\left[\mathrm{Si}O_{2}{\left(\mathrm{OH}\right)}_{2}\right]}^{2-}$ is varying with the continuous dosing of NaOH solution. The goodness of regression is highly dependent on the ratio of their concentration. So, parametrized concentration for normalization is not accessible. In addition, the quality of regression analysis is evaluated at different concentrations, especially at relatively high concentrations. Thus, the normalized deviation is not introduced in regression analysis.

The method for acquiring the optimized minimum R in group S0, as shown in Figure S.10, should be applicable for other measurements at different temperatures. But with the same method that was used to plot this figure, a minimum of R in the curve showing the R dependence of MSV at other temperatures has not been witnessed in group S1 to S7. The most possible reason is the low measuring sensitivity due to low concentration of silicate (this low sensitivity refers to the lower contribution of ${\left[\mathrm{SiO}{\left(\mathrm{OH}\right)}_{3}\right]}^{-}$ and ${\left[\mathrm{Si}O_{2}{\left(\mathrm{OH}\right)}_{2}\right]}^{2-}$ to the conductivity property of the solution, compared with $Na^{+}$ and $OH^{-}$. This sensitivity does not concern the adopted techniques for measuring conductivity). While in group S0, the concentrations of the two main monomeric species are so high that polymerization starts. The same argument also explains the failure of the methods of directly solving overdetermined equation groups. If the sensitivity were increased by redoubling the amount of sodium silicate solid, $\lambda_{{\left[\mathrm{SiO}{\left(\mathrm{OH}\right)}_{3}\right]}^{-}\;}$ and $\lambda_{1/2\;{\left[\mathrm{Si}O_{2}{\left(\mathrm{OH}\right)}_{2}\right]}^{2-}}$ could be calculated by solving every two-equation combination in the overdetermined equation groups. The best solution would be acquired by optimizing the results. However, the initial concentration of silicate (5 mmol/kg in present work) is already the highest accessible value in C-S-H nucleation experiments, considering temperature range, feasibility of pH adjustment, rate of phase transformations of precursors of C-S-H, sensitivity of ion-selective-membrane of sodium and composition of pore solution in real hydration situation.

In group S1–S7, before the dosing of NaOH solution, ${\left[\mathrm{SiO}{\left(\mathrm{OH}\right)}_{3}\right]}^{-}$ is the most abundant species in solution. Even if $\lambda_{{\left[\mathrm{SiO}{\left(\mathrm{OH}\right)}_{3}\right]}^{-}}$ is calculated in each group of experiments in priority by setting $c_{{\left[\mathrm{Si}O_{2}{\left(\mathrm{OH}\right)}_{2}\right]}^{2-}}=0$, it is not possible to achieve a better regression result. ‘lsqlin’ solves for $\lambda_{{\left[\mathrm{SiO}{\left(\mathrm{OH}\right)}_{3}\right]}^{-}\;}$ and $\lambda_{1/2\;{\left[\mathrm{Si}O_{2}{\left(\mathrm{OH}\right)}_{2}\right]}^{2-}}$ at the same time and gives a solution with the minimum global deviation. $\lambda_{{\left[\mathrm{SiO}{\left(\mathrm{OH}\right)}_{3}\right]}^{-}\;}$ calculated via only one equation leads to higher deviation, compared with current regression method.

### 5.2. Experimental Error

A thin needle for lumbar puncture is used for dosing NaOH solution, instead of an anti-diffusion tip. As a result, it is inevitable that a small amount of NaOH will continuously leak into the solution. To quantify its effects, the stirring speed is adjusted to maximum. It takes about 2 min for the potentiostat to record 60 points from 1 MHz to 1 Hz. During four repeated measurements, the increase in solution resistance has not been witnessed even at 308.45 K. It means the experimental error due to diffusion from the open tip is negligible.

In all the experimental processes, the impact of carbonation has been minimized as much as possible. The conductivity deviation is shown in Figure 4; thus, it should not be attributed independently to carbonation. Figure S.12 presents the conductivity of water saturated with atmospheric $CO_{2}$ at different temperatures [12]. At 298.55 K and 303.45 K, the impact of carbonation is obviously higher than regression deviations (as shown in Table 5). In addition, the durations of cell calibration with KCl solution are controlled to be identical with other conductivity measurements. With a proper selection of cell constant, the calibrated conductivity fits McCleskey’s model very well where the effects of $CO_{2}$ have been carefully subtracted [49].

### 5.3. Limitation of the Superposition of Conductivity Model for Mixed Electrolytes

As discussed above, the principle of conductivity superposition only predicts correctly in infinite diluted solution, or in mixed electrolytes where the concentration of one component is much less than the background electrolyte (e.g., NaOH), as shown in Section 4.2. Although this principle is applicable for the investigation of phase transformation of C-S-H, it requires a more sophisticated theory to model the conductivity of pore solutions under real hydration environments. A compromise approach is to replace Kohlrausch’s law of the independent migration of ions with other models, e.g., the RS model.

## 6. Conclusions

Significant inconsistencies have been presented in published diffusion coefficients or the equivalent conductivities of two main monomeric silicate aqueous ions, ${\left[\mathrm{SiO}{\left(\mathrm{OH}\right)}_{3}\right]}^{-}$ and ${\left[\mathrm{Si}O_{2}{\left(\mathrm{OH}\right)}_{2}\right]}^{2-}$, which undoubtedly hindered the accuracy in the investigations of physico-chemical properties of silicate materials in electrolyte solutions. Aiming to eliminate ambiguity, the current work measured the equivalent conductivity of the two species via EIS. The following conclusions have been reached:

(1) EIS spectrum is an efficient and precise tool to measure the conductivity of aqueous solutions at specific temperatures (277.65 K to 308.45 K), without the facilitation of equivalent circuits.
(2) The conductivity estimated by McCleskey’s conductivity model for NaOH, proposed in 2011 [49], excesses the published data and the one deduced from Robinson–Stokes conductivity model at different temperatures (278.15 K to 303.15 K). It is found that in a mixed electrolyte solution, if the concentration of a composition is much less than the rest, the bulk electrical conductivity can be calculated by the superposition of the contribution of this composition and the conductivity of the background electrolytes. The conductivity of this composition can be calculated by Kohlrausch’s law of the independent migration of ions. The calibrated McCleskey’s conductivity model for NaOH shows high accuracy. The conductivity model for mixed electrolytes (Equation (11)) closely matches measured conductivities of $\mathrm{NaOH}\text{-}H_{2}O$ solutions and $\mathrm{NaOH}\text{-}Na_{2}CO_{3}\text{-}H_{2}O$ solutions at investigated temperature and concentration range.
(3) With the established model (Equation (11)), the equivalent conductivity of ${\left[\mathrm{SiO}{\left(\mathrm{OH}\right)}_{3}\right]}^{-}$ and ${\left[\mathrm{Si}O_{2}{\left(\mathrm{OH}\right)}_{2}\right]}^{2-}$ were calculated with the least-square method. Their temperature dependence (277.85 K to 308.45 K) can be, respectively, approximated to Equations (18) and (19). The maximum deviation of conductivity in $\mathrm{NaOH}\text{-}Na_{2}\mathrm{Si}O_{3}\text{-}H_{2}O$ aqueous solutions is ±0.265 mS/cm.

The new parameters measured in the current work would further advance the understanding of multiple physico-chemical phenomena of silicate materials in electrolyte solutions. For instance, in the study of phase transformations of C-S-H, the potentiometric method has been widely adopted to measure binodal and spinodal limits [63]. Accuracy of the measurements depends on the calculation of the junction potential of the calcium ion-selective electrode (ISE). The presented $\lambda_{{\left[\mathrm{SiO}{\left(\mathrm{OH}\right)}_{3}\right]}^{-}}$ and ${\;\lambda }_{1/2\;{\left[\mathrm{Si}O_{2}{\left(\mathrm{OH}\right)}_{2}\right]}^{2-}}$ make it feasible to calculate this potential. Also, based on mass balance, combining with electrical potentials measured by different ISEs, the temperature dependence of these two parameters allows to infer the composition of intermediates formed during binodal demixing with conductivity measurements. In addition, the self-diffusion coefficients of ${\left[\mathrm{SiO}{\left(\mathrm{OH}\right)}_{3}\right]}^{-}$ and ${\left[\mathrm{Si}O_{2}{\left(\mathrm{OH}\right)}_{2}\right]}^{2-}$ calculated via the Nernst–Einstein equation are key parameters in simulations of diffusion and adsorption in the vicinity of interfaces between silicate minerals and aqueous solutions. For more concentrated mixed electrolyte solutions, Equation (11) may not be applicable. A solution is to replace Kohlrausch’s law of the independent migration of ions in Equation (11) with other models, e.g., the RS model. The accuracy of the measurements can be further improved by the calibration of the parameter R in regression analysis at different temperatures.

## Figures and Tables

**Table 1:** Experimental substances descriptions.

Substance	IUPAC Name	$\mathrm{Molecular}\;\mathrm{Weight}/g\;mol^{-1}$	CAS Registry Number	Origin	Purity	Method of Purity Determination
$Na_{2}CO_{3}$	Sodium carbonate	105.99	497-19-8	Merck KGaA (Darmstadt, Germany)	>99.9% [24]	Acidimetric method (calculated on dried substance).
$Na_{2}\mathrm{Si}O_{3}\cdot 5H_{2}O$	Sodium metasilicate pentahydrate	212.14	10213-79-3	VWR chemicals (Leuven, Belgium)	>98.08%	Titrimetric method, showed in ISO 1692 [25] and ISO 1690 [26].
NaOH	Sodium hydroxide solution (1.04 mol/kg)	40.00	1310-72-3	Merck KGaA	99.5% to 100.5% (±0.3%) [27]	Volumetric titration at 293.15 K
KCl	Potassium chloride solution (3.31 mol/kg)	74.55	7447-40-7	Metrohm (Herisau, Switzerland)	100.1% *	Potentiometric measurement at 298.0 ± 0.2 K

* The standard uncertainty, μ=1.2%, is calculated via type B evaluation given in [28], and based on potentiometric measurement with a nominal value of 10.0 ± 3.0 mv and a measured value of 10.2 mv. The extended Debye–Hückel equation (Helgeson) was used to calculated the activity coefficients of $K^{+}$ [29]. The electrical potential of an ion-selective electrode for $Cl^{-}$ is correlated to ionic activity with the extended Nikolskii–Eisenman (N–E) equation [30].

**Table 2:** Composition of each experimental solution.

No.	T/K	$w_{i}/g$			$v_{i}/\mathrm{mL}$	
		$H_{2}O$	$Na_{2}CO_{3}$	$Na_{2}\mathrm{Si}O_{4}\cdot 5H_{2}O$	NaOH	KCl
K1	277.65	33.2542	0	0	0	0.010, 0.030, 0.050, 0.100, 0.150, 0.250, 0.400
K2	293.55	33.2171	0	0	0	0.080, 0.150, 0.250, 0.300, 0.600, 1.000, 1.300, 1.600, 2.000
K3	308.45	33.2282	0	0	0	0.150, 0.300, 0.700, 1.000, 1.300, 2.000, 3.000, 6.000, 10.000
N1	277.65	33.0969	0	0	0.050, 0.100, 0.150, 0.250, 0.500, 0.750	0
N2	282.55	33.2010	0	0	0.100, 0.250, 0.500, 0.750, 1.000	0
N3	287.55	33.3148	0	0	0.100, 0.250, 0.500, 0.750, 1.000, 2.000	0
N4	293.55	33.2000	0	0	0.250, 0.500, 0.750, 1.000, 2.000	0
N5	298.55	33.0630	0	0	0.250, 0.500, 0.750, 1.000, 2.000	0
N6	303.45	33.2332	0	0	0.500, 0.750, 1.000, 2.000, 3.000, 4.000	0
N7	308.15	33.2820	0	0	0.750, 1.000, 2.000, 3.000, 4.000	0
C1	277.65	33.2691	0.0178	0	0.050, 0.100, 0.150, 0.250, 0.500, 0.750, 1.000	0
C2	282.65	33.2604	0.0176	0	0.100, 0.250, 0.501, 0.750, 1.000, 2.000	0
C3	293.55	33.2553	0.0176	0	0.250, 0.500, 0.750, 1.000, 2.000, 3.000	0
C4	308.45	33.2682	0.0179	0	0.750, 1.000, 2.000, 3.000, 4.000, 6.000	0
S0	298.55	33.2507	0	0.0142, 0.0394, 0.0630, 0.0838, 0.1081	0	0
S1	277.85	33.2713	0	0.0352	0.050, 0.100, 0.150, 0.250, 0.500, 0.750	0
S2	282.65	33.0836	0	0.0352	0.100, 0.250, 0.500, 0.750, 1.000	0
S3	287.65	33.1705	0	0.0352	0.100, 0.250, 0.500, 0.750, 1.000, 2.000	0
S4	293.55	33.2451	0	0.0352	0.250, 0.500, 0.750, 1.000, 2.000	0
S5	298.55	33.2514	0	0.0352	0.250, 0.500, 0.750, 1.000, 2.000	0
S6	303.45	32.7884	0	0.0352	0.500, 0.750, 1.000, 2.000, 3.000, 4.000	0
S7	308.45	33.2020	0	0.0352	0.750, 1.000, 2.000, 3.000, 4.000	0

$w_{i}$ refers to the total weight of solids. $v_{i}$ indicates the total volume of discrete added solutions. The accuracy of temperature electrode is 0.1 K. The standard uncertainty, μ(T) = 0.031 K, is calculated via Type A evaluation [28]. The accuracy of analytical balance is 0.0001 g. The standard uncertainty, $\mu (w_{i})$ = 0.00029 g, is calculated via Type B evaluation [28]. The standard uncertainty, $\mu (v_{i})$ = 0.14 μL, is calculated via Type A evaluation [28] by measuring the weight of 0.5, 1, 1.5, and 2 mL 0.1 mol/L NaCl standard solution (purchased from VWR chemicals) at 298.15 K.

**Table 3:** Correction for McCleskey’s conductivity model for NaOH aqueous solution.

T/K	278.15	283.15	288.15	293.15	298.15	303.15	308.15
$c_{\mathrm{NaOH}}\cdot \Lambda_{\mathrm{calibrated}}/\mathrm{mS}\;cm^{-1}$	0.975$\Lambda_{m}$	0.975$\Lambda_{m}$	$0.975\;(\Lambda_{m}-\;0.040)$	0.980$\Lambda_{m}$	0.982 ($\Lambda_{m}-\;0.050$)	0.980$\Lambda_{m}$	$\Lambda_{m}$
$R^{2}$	0.9993	0.9999	0.9999	0.9925	0.9999	1.0000	0.9999

**Table 4:** Part of the periodic table of elements with equivalent ionic conductance at 273.15 K. The bracketed numbers after element names indicate the ratio of equivalent conductivity of the double charged anion and the single charged one, R.

C (1.56)		N		O		F
${\left[CO_{2}\left(\mathrm{OH}\right)\right]}^{-}$ 44.50 $S\;cm^{2}\;mol^{-1}$	12CO32- 69.30 $S\;cm^{2}\;mol^{-1}$	/	/	/	/	/
**Si**		**P (1.58)**		**S (1.54)**		**Cl**
${\left[\mathrm{SiO}{\left(\mathrm{OH}\right)}_{3}\right]}^{-}$	12 SiO2OH22-	${\left[{\mathrm{PO}}_{2}{\left(\mathrm{OH}\right)}_{2}\right]}^{-}$	12 PO3OH2-	${\left[{\mathrm{SO}}_{3}\left(\mathrm{OH}\right)\right]}^{-}$	12 SO42-	${\left[\mathrm{Cl}O_{4}\right]}^{-}$
Tetrahedral,		Tetrahedral,		Tetrahedral,	Tetrahedral,	Tetrahedral,
Si-O 162 pm				S-O 149 pm		Cl-O 144 pm
		36.00 $S\;cm^{2}\;\mathrm{mo}l^{-1}$	57.00 $S\;cm^{2}\;\mathrm{mo}l^{-1}$	52.00 $S\;cm^{2}\;\mathrm{mo}l^{-1}$	80.00 $S\;cm^{2}\;\mathrm{mo}l^{-1}$	67.30 $S\;cm^{2}\;\mathrm{mo}l^{-1}$

**Table 5:** Equivalent conductivity of ${\left[\mathrm{SiO}{\left(\mathrm{OH}\right)}_{3}\right]}^{-}$ and ${\left[\mathrm{Si}O_{2}{\left(\mathrm{OH}\right)}_{2}\right]}^{2-}$ in aqueous solution at 277.85 K, 282.65 K, 287.65 K, 293.65 K, 298.55 K, 303.45 K, and 308.45 K.

T/K	277.85	282.65	287.65	293.65	298.55	303.45	308.45
$\lambda_{{\left[\mathrm{SiO}{\left(\mathrm{OH}\right)}_{3}\right]}^{-}}/S\;cm^{2}\;\mathrm{mo}l^{-1}$	12.66	14.03	17.01	18.41	21.20	24.09	26.03
$\lambda_{{1/2\;\left[\mathrm{Si}O_{2}{\left(\mathrm{OH}\right)}_{2}\right]}^{2-}}/S\;cm^{2}\;\mathrm{mo}l^{-1}$	19.78	21.92	26.57	28.77	33.12	37.64	40.66
${\left(\mathrm{MSV}\right)}^{0.5}/S\;cm^{2}$	±0.048	±0.03	±0.061	±0.157	±0.093	±0.065	±0.265

## Data Availability

The original contributions presented in this study are included in the article/Supplementary Materials. Further inquiries can be directed to the corresponding author.

## Supplementary Materials

The following supporting information can be downloaded at: https://www.mdpi.com/article/10.3390/ma18132996/s1, Figure S.1: Experimental setup which is composed of a titration unit, a jacketed reactor, EIS measuring unit and a temperature control unit. Figure S.2: Molality, $m_{\mathrm{KCl}}$, and density, $\rho_{\mathrm{KCl}}$, for KCl aqueous solution, as function of molarity, $c_{\mathrm{KCl}}.$ ∆, $\rho_{\mathrm{KCl}}$; **○**, $m_{\mathrm{KCl}}$; —, Equation (S-14); …, Equation (S-15). Figure S.3: Molality, $m_{\mathrm{NaOH}}$, and density, $\rho_{\mathrm{NaOH}}$, for NaOH aqueous solution, as function of molarity, $c_{\mathrm{NaOH}}.$ ∆, $\rho_{\mathrm{NaOH}}$; **○**, $m_{\mathrm{NaOH}}$; —, Equation (S-11); …, Equation (S-12). Figure S.4: Comparison of published conductivity data and McCleskey’s model for KCl aqueous solution at: —, 278.15 K; **- - -**, 293.15 K; and —·—, 308.15 K. □, measured data at 278.15 K [12]; +, measured at 278.15 K [49]; ∆, measured at 293.15 K [12]; ◊, measured at 293.15 K [8]; ○, measured at 308.15 K [12]; x, measured at 308.15 K [8]; ж, at 308.15 K. Figure S.5: Calibration with KCl solution at 277.65 K. □, measured conductivity with $C=1.055\;cm^{\text{-}1}$ (R^2^ = 0.9998). —, Equation (11). ○ refers to cell constant calculated by ratio between the conductivity calculated from Equation (11) and directly measured value. Figure S.6: Calibration with KCl solution at 293.55 K. □, measured conductivity with $C=1.055\;cm^{\text{-}1}$ (R^2^ = 0.9999). —, Equation (11). ○ refers to cell constant calculated by ratio between the conductivity calculated from Equation (11) and directly measured value. Figure S.7: Calibration with KCl solution at 308.45 K. □, measured conductivity with $C=1.055\;cm^{\text{-}1}$ (R^2^ = 0.9972). —, Equation (11). ○ refers to cell constant calculated by ratio between the conductivity calculated from Equation (11) and directly measured value. Figure S.8: Evaluation of cell constant, C, with the coefficient of determination, $R^{2}$. …, 277.65 K; —, 293.55 K; ---, 308.45 K. Figure S.9: Verification of calibrated McCleskey’s conductivity model for NaOH with conductivities measured at 277.65 K (a), 282.55 K (b), 287.55 K (c), 293.55 K (d), 298.55 K (e), 303.45 K (f) and 308.15 K (g). x, measured conductivities; Equation (11). Figure S.10: R dependence of mean square variance, MSV, of the equivalent conductivity of ${\left[\mathrm{SiO}{\left(\mathrm{OH}\right)}_{3}\right]}^{-}$ and ${\left[\mathrm{Si}O_{2}{\left(\mathrm{OH}\right)}_{2}\right]}^{2-}$ ions of group S0 at 298.55 K. Figure S.11: R dependence of averaged mean square variance, (MSV) and MSV of the maximum deviation (dev) of the equivalent conductivity of ${\left[\mathrm{SiO}{\left(\mathrm{OH}\right)}_{3}\right]}^{-}$ and ${\left[\mathrm{Si}O_{2}{\left(\mathrm{OH}\right)}_{2}\right]}^{2\text{-}}$ ions of group S1 to S6. Figure S.12: Conductivity of water saturated with atmospheric $CO_{2}$ at different temperatures. Table S.1: Reported diffusion coefficient ($D_{i}$) and equivalent conductivity ($\lambda_{i}$) of silicate species in aqueous solution. Table S.2: Thermophysical properties of pure water at temperature range of 273.16 K to 313.15 K. Table S.3: Values of $b_{\gamma }$ and $\dot{a}$ for calculation in different background solutions. Table S.4: Standard (partial molal) thermodynamic properties and equation of state parameters of aqueous species at 298.15 K, 1 bar used in GEMS3 calculations. Table S.5: Composition, measured conductivities, and uncertainties of KCl solutions at 277.65 K, 293.55 K, and 308.45 K. Table S.6: Concentrative properties (molality, molarity, density) of KCl, NaOH aqueous solutions at 293.15 K. Table S.7: Composition, measured conductivities, and uncertainties of NaOH solutions at 277.65 K, 282.55 K, 287.55 K, 293.55 K, 298.55 K, 303.45 K and 308.15 K. Table S.8: Composition and measured conductivity of $Na_{2}CO_{3}\;+\;\mathrm{NaOH}$ solutions at 277.65 K, 282.55 K, 293.55 K, and 308.45 K. Table S.9: Uncertainties of the composition and measured conductivity of $Na_{2}CO_{3}\;+\;\mathrm{NaOH}$ solutions at 277.65 K, 282.55 K, 293.55 K, and 308.45 K. Table S.10: Composition and measured conductivity of $\mathrm{NaOH}\text{-}Na_{2}\mathrm{Si}O_{3}\text{-}H_{2}O$ solutions at 277.85 K, 282.65 K, 287.65 K,293.65 K, 298.55 K, 303.45 K, and 308.45 K. Table S.11: Uncertainties of the composition and measured conductivity of $\mathrm{NaOH}\text{-}Na_{2}\mathrm{Si}O_{3}\text{-}H_{2}O$ solutions at 277.85 K, 282.65 K, 287.65 K,293.65 K, 298.55 K, 303.45 K, and 308.45 K. References [8,11,12,13,14,15,16,17,28,29,40,49,61,64,65,66,67,68,69] have been cited in the Supplementary Materials.
