# Supplementary material for: Study and Evaluation of Equivalent Conductivities of [SiO(OH)3]− and [SiO2(OH)2]2− in NaOH-Na2SiO3-H2O Solutions at 277.85 K to 308.45 K
Source: Materials (Basel). 2025 Jun 24;18(13):2996. doi: 10.3390/ma18132996 (PMC12251526; doi:10.3390/ma18132996)
Supplement: Supplementary file 1 [file materials-18-02996-s001.zip › materials-3684744-supplementary.pdf]

Supplementary Information

Study and Evaluation of Equivalent Conductivities of $[SiO(OH)_3]^-$ and $[SiO_2(OH)_2]^{2-}$ in NaOH-Na_2SiO_3-H_2O Solutions at 277.85 K to 308.45 K

Kai Yang [1], Guang Ye [2] and Geert De Schutter [1,*]

[1] Department of Structural Engineering and Building Materials, Ghent University, B-9052 Ghent, Belgium; kai.yang@ugent.be
[2] Department of Materials and Environment (Microlab), Delft University of Technology, 2628 CN Delft, The Netherlands; g.ye@tudelft.nl
* Correspondence: geert.deschutter@ugent.be

1. Experimental setup

Figure S. 1 demonstrated the simplified experimental setup.

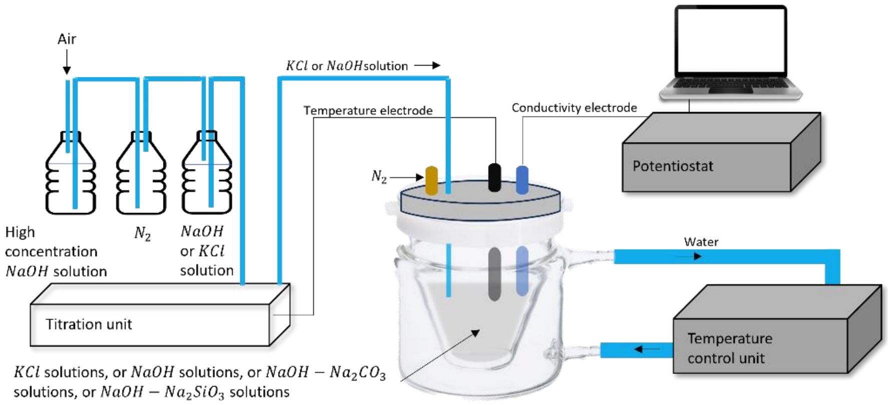

Figure S. 1. Experimental setup which is composed of a titration unit, a jacketed reactor, EIS measuring unit and a temperature control unit.

2. Diffusion coefficients for aqueous silicate species in literature

Nerst-Einstein equation, equation S-1, correlates the self-diffusion coefficient of the ions, D_i (in $m^2\ s^{-1}$), with λ_i without considering the electrical neutrals:

$$D_i = \frac{RT\lambda_i}{z_i^2 F^2} \qquad (S\text{-}1)$$

where R is the gas constant (expressed in $J\ mol^{-1}\ K^{-1}$), T is temperature in Kelvin (K), and F is the Faraday constant (expressed in $C\ mol^{-1}$) [40, 13]. Table S. 1 compares D_i of specific silicate species in aqueous solutions in related published research and measured value of $\lambda_{[SiO_2(OH)_2]^{2-}}$.

Table S. 1. Reported diffusion coefficient (D_i) and equivalent conductivity (λ_i) of silicate species in aqueous solution.

Authors	T/K	$D_i/10^{-9}\ m^2\ s^{-1}$		$\lambda_i/S\ cm^2\ mol^{-1}$		Method
Yokoyama [15]	298.15	$D_{[Si(OH)_4]} \approx D_{[SiO(OH)_3]^-}$	0.0194 to 0.0197			Time dependence of concentration variation
Applin [64]	298.65 ± 0.5	$D_{[Si(OH)_4]}$	2.2			
		$D_{[(HO)_3SiOSi(OH)_3]}$	1.0			
Pilson [65]	278.15	D_{SiO_2}	0.33			
House, et al. [66]	281.95	D_{SiO_2}	1.21			
Rebreanu, et al. [14]	275.15 to 303.15	D_{SiO_2}	0.47 to 1.17			Conductivity
Qiu, et al. [16] and Huang [17]	298.15	$D_{[SiO_2(OH)_2]^{2-}}$	0.7			
Greenberg [61]	298.15			$\lambda_{[SiO_2(OH)_2]^{-}}$	35	Conductivity

3. Thermodynamic calculation for ionic activity and solution composition.

The ionic activity coefficients were calculated using Debye-Hückel equation (Helgeson) [29], as shown below:

$$\log_{10}\gamma_j = \frac{-A_\gamma z_j^2\sqrt{I}}{1+\dot{a}B_\gamma\sqrt{I}} + b_\gamma I + \log_{10}\frac{x_{jw}}{X_w} \qquad (S\text{-}2)$$

There, γ_j is activity coefficient of j-th species, z_j is the formula charge, I is the effective ionic strength, ȧ (in 10^{-8} cm) is an average distance of approach of two ions of opposite charge, b_γ is a semi-empirical coefficient, x_{jw} is the mole quantity of water-solvent, X_w is the total mole quantity of the aqueous phase (including water-solvent), and

$$A_\gamma=1.82483\cdot10^6\rho_0^{0.5}(\varepsilon_0\cdot T)^{-1.5} \qquad (S\text{-}3)$$

$$B_\gamma=50.2916\rho_0^{0.5}(\varepsilon_0\cdot T)^{-0.5} \qquad (S\text{-}4)$$

$$I=\frac{1}{2}\sum_j^{n(L_w)} m_j z_j^2 \qquad (S\text{-}5)$$

Where ρ_0 is density ($g\cdot cm^{-3}$) of pure water at temperature T. The corresponding properties of water are shown in Table S.2. [12] presents the T dependence of ρ_0, every 0.1 K from 273.25 K to 321.15 K, which has been fitted to equation S-6. Besides, ε_0 at investigated T can be obtained from equation S-7. $n(L_w)$ is the kind of aqueous species, m_j is the molality of j-th species (in moles per 1 kg H_2O). The last term in equation S-2 is used to convert the activity coefficient calculated from mole fraction scale into practical (molality) scale. R^2 is the coefficient of determination, RSS is sum of squares of residauals, TSS is total sum of squares.

Table S. 2. Thermophysical properties of pure water at temperature range of 273.16 K to 313.15 K.

T/K	$\rho_0/g\cdot cm^{-3}$	ε_0	η/μPa s
273.16	0.99984	87.899	1791.1
283.15	0.9997	83.974	1305.9
293.15	0.99821	80.223	1001.6
298.15	0.99705	78.408	890.02
303.15	0.99565	76.634	797.22
313.15	0.99222	73.201	652.73

$$\rho_0=-3.7754\times10^{-10}T^4+7.0237\times10^{-8}T^3-8.5949\times10^{-6}T^2+6.4837\times10^{-5}T+0.9985,\ R^2=1 \qquad (S\text{-}6)$$

$$\varepsilon_0=7.2109\times10^{-4}T^2-3.9504\times10^{-1}T^2+87.860,\ R^2=0.9999 \qquad (S\text{-}7)$$

$$R^2=1-\frac{RSS}{TSS} \qquad (S\text{-}8)$$

Table S. 3. Presents the values of ȧ and b_γ, introduced in thermodynamic calculations with GEMS3. In group K1 to K3, KCl solution was selected as background solution, while in other groups, the parameters for NaOH solutions were used.

Table S. 3. Values of b_γ and ȧ for calculation in different background solutions

Solution	NaCl	KCl	NaOH	KOH
b_γ	0.064	0.025	0.098	0.123
ȧ/10^{-8} cm	3.72	4.08	3.31	3.67

Table S. 4. Addresses other thermodynamic parameters to calculate composition of each solution with GEMS3 [11].

Table S. 4. Standard (partial molal) thermodynamic properties and equation of state parameters of aqueous species at 298.15 K, 1 bar used in GEMS3 calculations.

Species	NaOH@	$Na(CO_3)^-$	$[SiO(OH)_3]^-$	SiO_2@*		$[SiO_2(OH)_2]^{2-}$**		$[Si_4O_{10}]^{4-}$
ΔG^0/ kJ mol^{-1}	-418.12	-797.11	-1014.60		-833.41		-938.51	-3600.81
ΔH^0/ kJ mol^{-1}	-470.14	-938.56	-1144.68		-887.86		-1098.74	-3915.99
S^0/ J K mol^{-1}	44.77	-44.31	20.92		41.34		-80.20	305.20
Cp^0/ J K mol^{-1}	-13.40	-51.28	-87.20		44.47		119.83	328.58
V^0/ J bar^{-1}	3.51	-0.42	4.53		1.61		0	0
$a_1\cdot10$/cal mol^{-1}bar^{-1}	2.2338	2.3862	2.9735	a_0	46.64	A_0	-10.0006	0
$a_2\cdot10^{-2}$/cal mol^{-1}	-2.3287	-1.9521	-0.5181	a_1	0.034	A_1	0	0
a_3/cal K mol^{-1}bar^{-1}	6.6683	6.5103	5.9467	a_2	-1.13×10^6	A_2	-3917.5	-10822.8
$a_4\cdot10^{-4}$/cal K mol^{-1}	-2.6826	-2.6982	-2.7575					

c_1/cal mol^{-1}K^{-1}	4.0146	15.3395	8.1489
$c_2\ 10^{-4}$/cal K mol^{-1}	-3.6863	-5.5686	-7.3123
$\omega_0 \cdot 10^{-5}$/cal mol^{-1}	-0.0300	1.7870	1.5511

ΔG^0, ΔH^0, S^0, Cp^0, and V^0 are the standard Gibbs energies, enthalpy, entropy, heat capacity, and volume of elements, at 298.15 K respectively. a_1, a_2, a_3, a_4, c_1, c_2 and ω_0 are parameters defined in Helgeson-Kirkham-Flowers (HKF) equation of state, given in original calorimetric units [67, 68].

*Temperature correction using Cp(T) integration $Cp^0=a_0+a_1T+a_2T^{-2}$.

**Temperature correction using $\log K(T) = A_0+A_1T+A_2T^{-1}$.

4. Calibration for cell constant with KCl-H_2O solutions at different temperatures

The compositions of KCl solutions at 277.65 K, 293.55 K, and 308.45 K are shown in Table S. 5.

Table S. 5. Composition, measured conductivities, and uncertainties of KCl solutions at 277.65 K, 293.55 K, and 308.45 K.

T/K	w_i/g	v_i/mL	m_i/mol·kg^{-1} (water)	$u^2_{m_i}$/mol^2·kg^{-2} (water)	κ/mS·cm^{-1}	u(κ)/mS·cm^{-1}
	H_2O	KCl	K^+ or Cl^-	K^+ or Cl^-		
277.65	33.2542	0.010	0.0009026	1.593×10^{-10}	0.094	0.000134
		0.030	0.0027063	1.590×10^{-10}	0.259	0.000540
		0.050	0.0045081	1.587×10^{-10}	0.424	0.000541
		0.100	0.0090039	1.578×10^{-10}	0.816	0.002024
		0.150	0.0134874	1.569×10^{-10}	1.197	0.000774
		0.250	0.0224181	1.553×10^{-10}	1.943	0.002289
		0.400	0.0357235	1.528×10^{-10}	3.034	0.001725
293.55	33.2171	0.080	0.0072094	1.585×10^{-10}	0.967	0.000438
		0.150	0.0134919	1.573×10^{-10}	1.758	0.002184
		0.250	0.0224255	1.556×10^{-10}	2.851	0.005031
		0.300	0.0268742	1.548×10^{-10}	3.395	0.001359
		0.600	0.0533148	1.500×10^{-10}	6.531	0.001599
		1.000	0.0879125	1.441×10^{-10}	10.462	0.007933
		1.300	0.1133814	1.400×10^{-10}	13.288	0.005251
		1.600	0.1384502	1.361×10^{-10}	15.990	0.004737
		2.000	0.1712691	1.312×10^{-10}	19.449	0.012676
308.45	33.2282	0.150	0.0134874	1.572×10^{-10}	2.413	0.003366
		0.300	0.0268653	1.547×10^{-10}	4.651	0.001258
		0.700	0.0620134	1.484×10^{-10}	10.175	0.001596
		1.000	0.0878839	1.440×10^{-10}	14.107	0.005368
		1.300	0.1133448	1.399×10^{-10}	17.814	0.008801
		2.000	0.1712148	1.312×10^{-10}	25.954	0.004627
		3.000	0.2503377	1.207×10^{-10}	36.523	0.016103
		6.000	0.4654208	9.924×10^{-11}	70.452	0.011525
		10.000	0.7091250	8.443×10^{-11}	95.369	0.053786

The standard uncertainty: u(T) = 0.031 K, $u(w_i)$ = 0.00029 g, $u(v_i)$ = 0.14 µL. The calculation methods are identical to the ones shown in Table 2. The combined standard uncertainty $u^2_{m_i}=1.593\times10^{-10}$ mol^2·kg^{-2}, according to equations S-9 to S-13. The combined expanded uncertainty U is 0.275% with 0.9973 level of confidence (k=3). κ is arithmetic averaged conductivity of four repeatable measurements. u(κ) is calculated with Type A evaluation [28]. The maximum u(κ) is 0.053786 mS·cm^{-1}.

As the input quantities T, w_{H_2O}, and v_{KCl} are uncorrelated, the combined standard uncertainty $u^2_{m_{K^+}}$ and $u^2_{m_{Cl^-}}$ is obtained from the following equation [28,69]:

$$u^2_{m_i}=\left(\frac{\partial m_i}{\partial T}\right)^2 u^2(T)+\left(\frac{\partial m_i}{\partial w_i}\right)^2 u^2(w_i)+\left(\frac{\partial m_i}{\partial v_{KCl}}\right)^2 u^2(v_{KCl}) \quad (S\text{- }9)$$

And

$$m_{K^+}=m_{Cl^-}=\frac{\frac{v_{KCl}\cdot 3}{1000}}{\frac{\left(w_{H_2O}+v_{KCl}\cdot\rho_{KCl}\cdot\frac{v_{KCl}}{1000}\cdot 3\cdot M_{KCl}\right)}{1000}} \quad (S\text{- }10)$$

$$\frac{\partial m_{K^+}}{\partial T}=0 \quad (S\text{- }11)$$

$$\frac{\partial m_{K^+}}{\partial w_{H_2O}}=-\frac{3000000v_{KCl}}{\left(3v_{KCl}M_{KCl}\text{-}1000v_{KCl}\rho_{KCl}\text{-}1000w_{H_2O}\right)^2} \quad (S\text{- }12)$$

$$\frac{\partial m_{K^+}}{\partial v_{KCl}}=\frac{3000000w_{H_2O}}{\left(3v_{KCl}M_{KCl}\text{-}1000v_{KCl}\rho_{KCl}\text{-}1000w_{H_2O}\right)^2} \quad (S\text{- }13)$$

Where ρ_{KCl} indicates density of KCl solution (1.1314 g cm^{-3}, which calculated via correlating m_{KCl} and c_{KCl}, shown in Table S. 6. The fitting equation is equation S-14, which is also illustrated in Figure S.2.), M_{KCl} is the molecular weight of KCl (74.55 g mol^{-1}). As shown in equations S-9 and S-12, each v_i leads to a $u^2_{m_i}$. The final $u^2_{m_i}$ is presented by the maximum of $u^2_{m_i}$.

Table S. 6. Concentrative properties (molality, molarity, density) of KCl, NaOH aqueous solutions at 293.15 K [12].

m_i/mol·kg^{-1}		c_i/mol·L^{-1}		ρ_i/g cm^{-3}	
KCl	NaOH	KCl	NaOH	KCl	NaOH
0.067	0.126	0.067	0.125	1.0014	1.0039
0.135	0.253	0.135	0.252	1.0046	1.0095
0.274	0.51	0.271	0.51	1.011	1.0207
0.415	0.773	0.409	0.774	1.0174	1.0318
0.559	1.042	0.549	1.043	1.0239	1.0428
0.706	1.316	0.691	1.317	1.0304	1.0538
0.856	1.596	0.835	1.597	1.0369	1.0648
1.01	1.882	0.98	1.883	1.0434	1.0758
1.166	2.174	1.127	2.174	1.05	1.0869
1.327	2.473	1.276	2.47	1.0566	1.0979
1.49	2.778	1.426	2.772	1.0633	1.1089
1.658	3.09	1.579	3.08	1.07	1.1199
1.829	3.409	1.733	3.393	1.0768	1.1309
2.004	3.736	1.89	3.711	1.0836	1.1419
2.184	4.07	2.048	4.036	1.0905	1.153
2.367	4.412	2.208	4.365	1.0974	1.164
2.555	4.762	2.37	4.701	1.1043	1.1751
2.747	5.121	2.534	5.041	1.1114	1.1861
2.944	5.488	2.701	5.387	1.1185	1.1971
3.146	5.865	2.869	5.739	1.1256	1.2082
3.353	6.25	3.039	6.096	1.1328	1.2192
3.783	7.052	3.386	6.827	1.1474	1.2412
4.236	7.895	3.742	7.579	1.1623	1.2631

$$\rho_{KCl}=-0.0007c_{KCl}+0.0464c_{KCl}+0.9985,\ R^2=1.0000 \qquad \textit{(S- 14)}$$

$$m_{KCl}=0.0384c_{KCl}+0.9858c_{KCl}+0.0045,\ R^2=1.0000 \qquad \textit{(S- 15)}$$

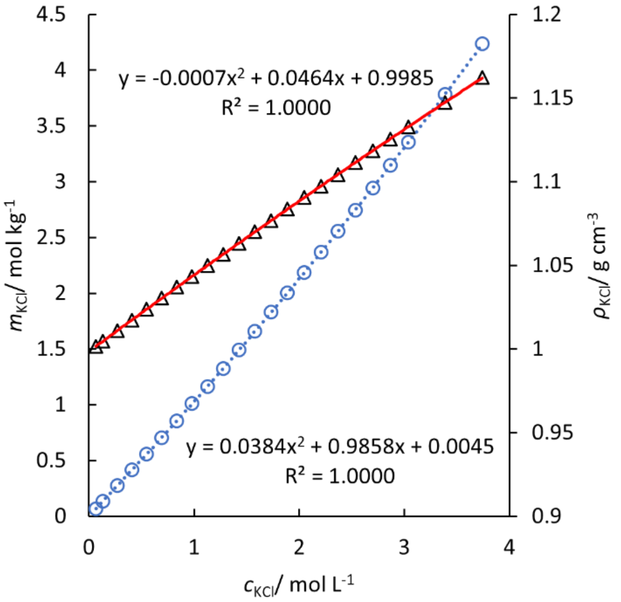

Figure S. 2. Molality, m_{KCl}, and density, ρ_{KCl}, for KCl aqueous solution, as function of molarity, c_{KCl}. Δ, ρ_{KCl}; ○, m_{KCl}; —, equation. S-14; …, equation. S-15.

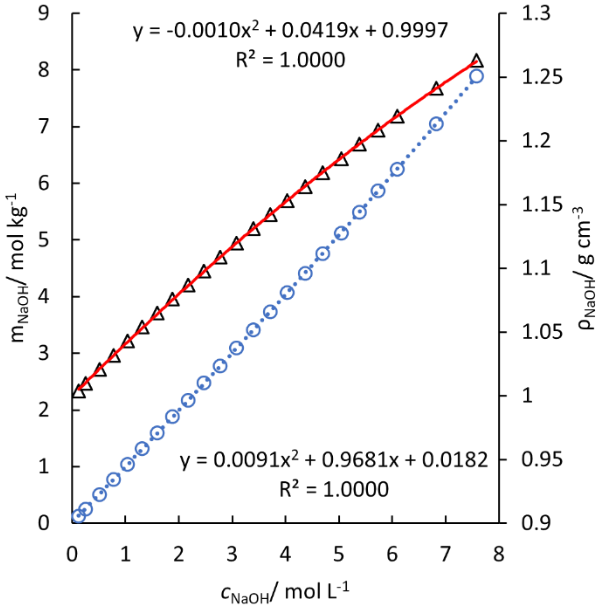

Figure S. 3. Molality, m_{NaOH}, and density, ρ_{NaOH}, for NaOH aqueous solution, as function of molarity, c_{NaOH}. Δ, ρ_{NaOH} ; ○, m_{NaOH} ; —, equation. S-11; …, equation. S-12.

With the same method, the molality, m_{NaOH}, and density, ρ_{NaOH}, of NaOH solution are fitted into equation S-16 and equation S-17, which are shown in Figure S. 3.

$$\rho_{NaOH}=-0.0010c_{NaOH}+0.0419c_{NaOH}+0.9997,\ R^2=1.0000 \qquad (S\text{- }16)$$

$$m_{NaOH}=0.0091c_{NaOH}+0.9681c_{NaOH}+0.0182,\ R^2=1.0000 \qquad (S\text{- }17)$$

Figure S. 4. Compares the conductivities predicted by McCleskey's model (equation 11 with equations S-18 to S-20 as input) and published data. The model coincides with experiments very well at 278.15 K, 293.15 K, and 308.15 K in indicated concentration range (lower than 1 mol kg^{-1}). Thus, in the following calibration, this model was taken as reference.

$$\Lambda_m^0(t)=0.009385t^2+2.533t+81.17 \qquad (S\text{- }18)$$

$$A=0.01390t^2+1.886t+56.76 \qquad (S\text{- }19)$$

$$B=1.7 \qquad (S\text{- }20)$$

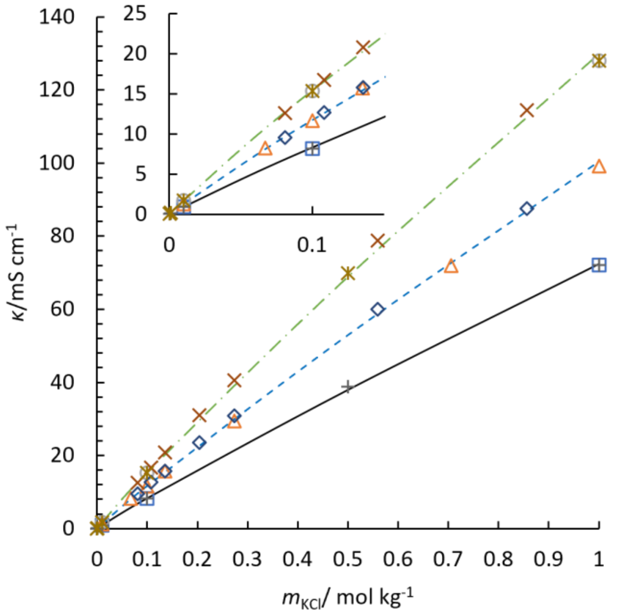

Figure S. 4. Comparison of published conductivity data and McCleskey's model for KCl aqueous solution at: —, 278.15 K; - - - , 293.15 K; and —·—, 308.15 K. □, measured data at 278.15 K [12]; + , measured at 278.15 K [49]; Δ, measured at 293.15 K [12]; ◊, measured at 293.15 K [8]; ○, measured at 308.15 K [12]; x, measured at 308.15 K [8]; ж, at 308.15 K.

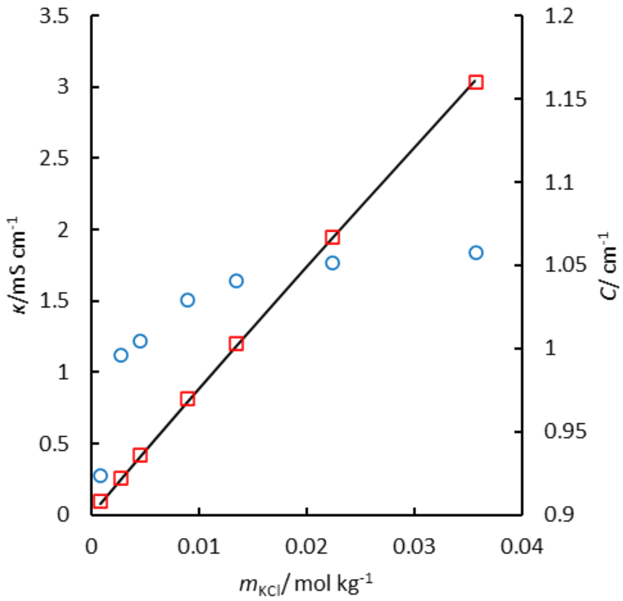

Figure S. 5. Calibration with KCl solution at 277.65 K. □, measured conductivity with C=1.055 cm^{-1} (R^2=0.9998). —, equation 11. ○ refers to cell constant calculated by ratio between the conductivity calculated from equation 11 and directly measured value.

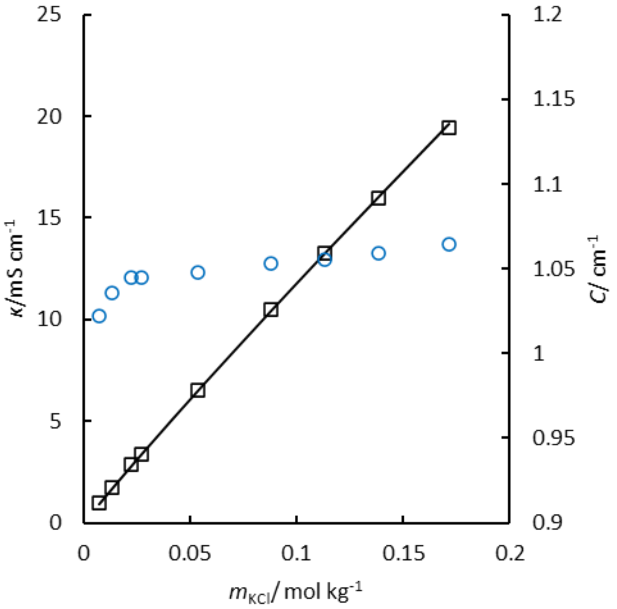

Figure S. 6. Calibration with KCl solution at 293.55 K. □, measured conductivity with C=1.055 cm^{-1} (R^2=0.9999). —, equation 11. ○ refers to cell constant calculated by ratio between the conductivity calculated from equation 11 and directly measured value.

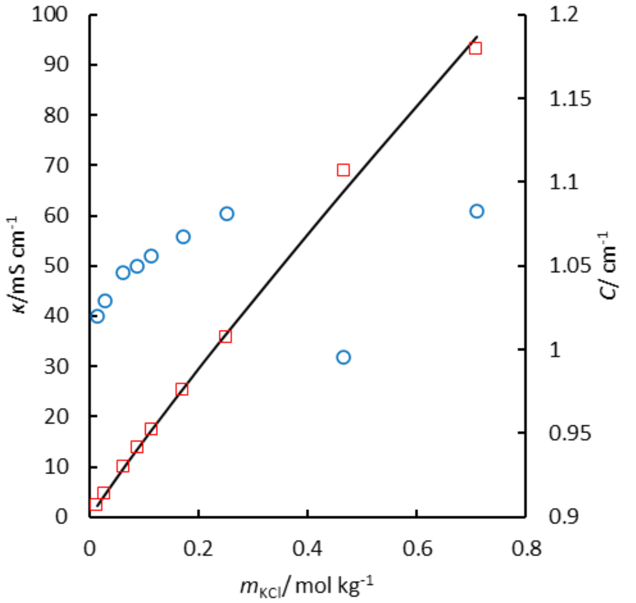

Figure S. 7. Calibration with KCl solution at 308.45 K. □, measured conductivity with C=1.055 cm^{-1} (R^2=0.9972). —, equation 11. ○ refers to cell constant calculated by ratio between the conductivity calculated from equation 11 and directly measured value.

The final cell constant, C, is determined by R^2. As shown in Figure S. 8, all the maximums of R^2at three different temperatures are witnessed at C=1.055 cm^{-1}. This value leads to high consistency between the calculated conductivity via equation 11 and measured data at 277.65 K (Figure S. 5.), 293.55 K (Figure S. 6.), and 308.45 K (Figure S. 7.). Therefore, this value is introduced in the following measurements.

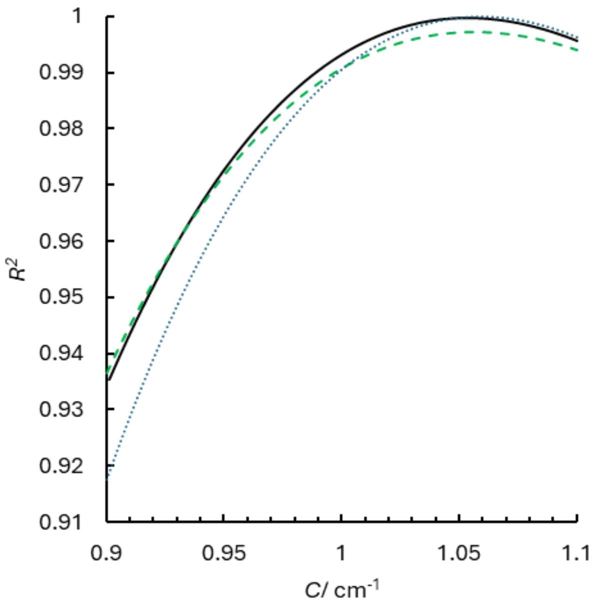

Figure S. 8. Evaluation of cell constant, C, with the coefficient of determination, R^2. ..., 277.65 K; —, 293.55 K; ---, 308.45 K.

5. Composition and conductivity of NaOH-H_2O solutions at different temperatures

Table S. 7. presents the compositions of NaOH-H_2O solutions and uncertainties.

Table S. 7. Composition, measured conductivities, and uncertainties of NaOH solutions at 277.65 K, 282.55 K, 287.55 K, 293.55 K, 298.55 K, 303.45 K and 308.15 K.

T/K	w_i/g	v_i/mL	m_i/mol·kg^{-1} (water)		$u^2_{m_i}$/mol·kg^{-1} (water)		κ/mS·cm^{-1}	u(κ)/mS·cm^{-1}
	H_2O	NaOH	NaOH@	Na^+ or OH^-	NaOH@	Na^+ or OH^-		
277.65	33.0969	0.050	0.0000014	0.0015070	7.791×10^{-16}	2.258×10^{-10}	0.250	0.000101
		0.100	0.0000055	0.0030069	1.257×10^{-14}	1.006×10^{-9}	0.491	0.000137
		0.150	0.0000119	0.0044998	6.063×10^{-14}	2.344×10^{-9}	0.722	0.000339
		0.250	0.0000317	0.0074653	4.394×10^{-13}	6.627×10^{-8}	1.190	0.001884
		0.500	0.0001163	0.0147659	6.011×10^{-12}	2.608×10^{-8}	2.326	0.002979
		0.750	0.0002448	0.0219136	2.194×10^{-11}	5.701×10^{-8}	3.435	0.001330
282.55	33.2010	0.100	0.0000054	0.0029976	2.959×10^{-15}	2.451×10^{-10}	0.544	0.000258
		0.250	0.0000310	0.0074426	1.099×10^{-13}	1.713×10^{-9}	1.328	0.002739
		0.500	0.0001139	0.0147224	1.751×10^{-12}	6.897×10^{-9}	2.603	0.001184
		0.750	0.0002398	0.0218507	7.312×10^{-12}	1.516×10^{-8}	3.834	0.003803
		1.000	0.0004030	0.0288356	1.498×10^{-11}	2.630×10^{-8}	5.041	0.001805
287.55	33.3148	0.100	0.0000053	0.0029874	1.967×10^{-16}	1.700×10^{-11}	0.616	0.000923
		0.250	0.0000305	0.0074178	5.164×10^{-15}	1.587×10^{-11}	1.497	0.001486
		0.500	0.0001120	0.0146744	2.237×10^{-14}	1.552×10^{-11}	2.928	0.001744
		0.750	0.0002358	0.0217809	2.664×10^{-14}	1.349×10^{-11}	4.317	0.002175
		1.000	0.0003964	0.0287452	2.046×10^{-13}	1.173×10^{-11}	5.655	0.000413
		2.000	0.0013316	0.0553006	4.587×10^{-13}	8.875×10^{-12}	10.630	0.001089
293.55	33.2000	0.250	0.0000305	0.0074433	1.073×10^{-13}	1.746×10^{-9}	1.673	0.002511
		0.500	0.0001119	0.0147248	1.382×10^{-12}	7.050×10^{-9}	3.265	0.003121
		0.750	0.0002356	0.0218556	5.262×10^{-12}	1.553×10^{-8}	4.814	0.001051
		1.000	0.0003955	0.0288439	1.235×10^{-11}	2.690×10^{-8}	6.306	0.001539
		2.000	0.0013292	0.0554877	1.980×10^{-10}	9.262×10^{-8}	11.825	0.004839
298.55	33.0630	0.250	0.0000307	0.0074739	5.466×10^{-13}	8.937×10^{-9}	1.844	0.008776
		0.500	0.0001126	0.0147847	7.847×10^{-12}	3.504×10^{-8}	3.604	0.000689
		0.750	0.0002368	0.0219438	3.048×10^{-11}	7.641×10^{-8}	5.296	0.002514
		1.000	0.0003978	0.0289592	8.226×10^{-11}	1.311×10^{-7}	6.939	0.007517
		2.000	0.0013337	0.0557052	8.324×10^{-10}	4.544×10^{-7}	13.054	0.002160

		0.500	0.0001110	0.0146753	2.681×10^{-14}	9.690×10^{-14}	3.892	0.004095
		0.750	0.0002336	0.0217831	3.793×10^{-14}	1.501×10^{-11}	5.719	0.003522
		1.000	0.0003925	0.0287491	1.528×10^{-13}	1.496×10^{-11}	7.496	0.002573
303.45	33.2332	2.000	0.0013160	0.0553162	4.300×10^{-13}	6.706×10^{-12}	14.105	0.003311
		3.000	0.0025853	0.0800229	3.876×10^{-13}	5.467×10^{-12}	20.105	0.009716
		4.000	0.0040975	0.1030940	1.162×10^{-13}	5.240×10^{-12}	25.616	0.005396
		0.750	0.0002345	0.0218034	1.153×10^{-14}	1.703×10^{-11}	6.308	0.007753
		1.000	0.0003936	0.0287759	1.354×10^{-13}	1.334×10^{-11}	8.239	0.002193
308.15	33.2820	2.000	0.0013192	0.0553657	1.571×10^{-12}	9.249×10^{-12}	15.511	0.011640
		3.000	0.0025905	0.0800924	8.739×10^{-13}	8.472×10^{-12}	22.127	0.011216
		4.000	0.0041048	0.1031810	2.297×10^{-12}	7.890×10^{-12}	28.188	0.011103

The standard uncertainty: u(T) = 0.031 K, u(w_i) = 0.00029 g, u(v_i) = 0.14 μL. The calculation methods are identical to the ones shown in Table 2. The maximum of combined standard uncertainty $u^2_{m_{NaOH@}}$=1.980×10^{-10} mol^2·kg^{-2}, $u^2_{m_{Na^+}}$=4.544×10^{-7} mol^2·kg^{-2}. The combined expanded uncertainty U is 0.109 % with 0.9973 level of confidence (k=3). κ is arithmetic average of four repeatable measurements. u(κ) is calculated with Type A evaluation [28]. The maximum u(κ) is 0.011216 mS·cm^{-1}.

Figure S. 9. compares the calibrated model, equation 11, with measured conductivities of NaOH-H_2O solutions at 277.65 K to 308.15 K.

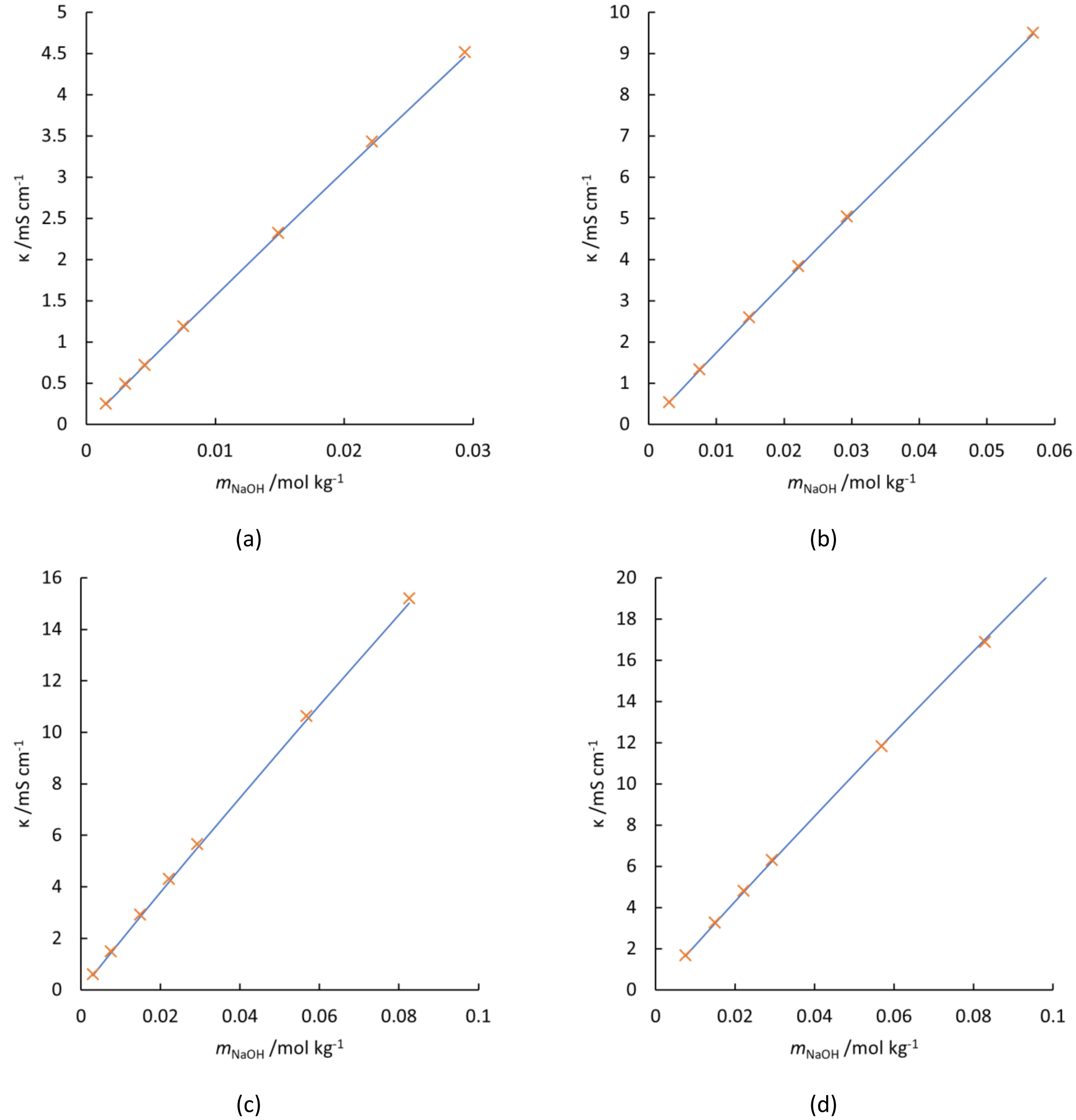

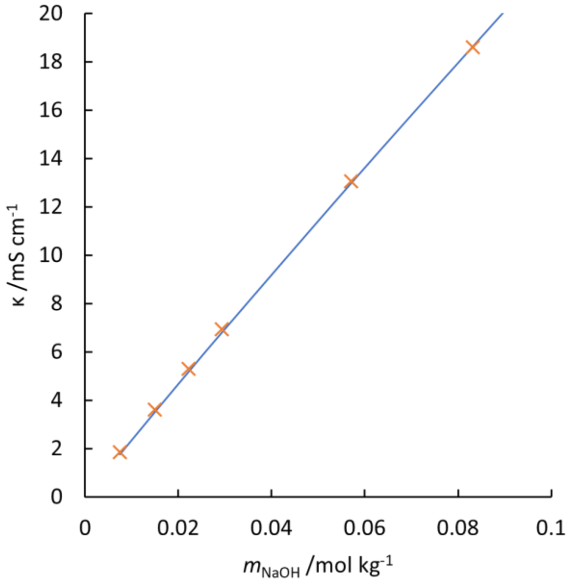

(e)

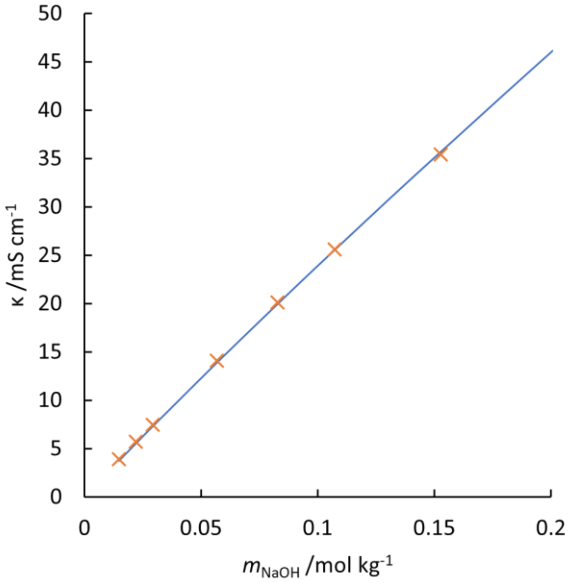

(f)

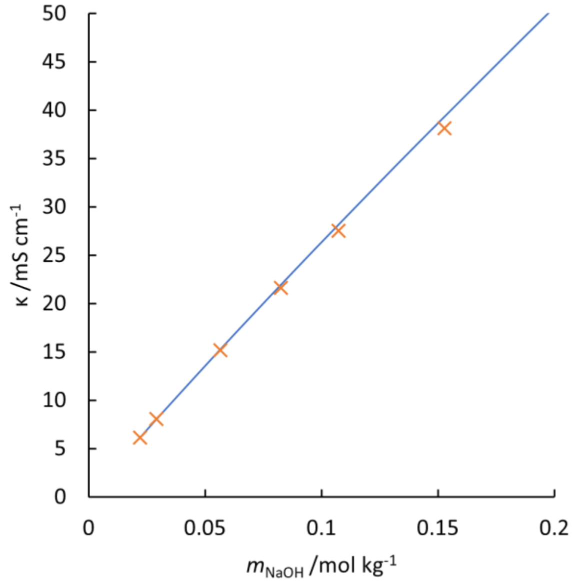

(g)

Figure S. 9. Verification of calibrated McCleskey's conductivity model for NaOH with conductivities measured at 277.65 K (a), 282.55 K (b), 287.55 K (c), 293.55 K (d), 298.55 K (e), 303.45 K (f) and 308.15 K (g). x, measured conductivities; —, equation 11.

6. Composition and conductivity of $NaOH$-Na_2CO_3-H_2O solutions at different temperature

Table S. 8. presents the compositions and measured conductivities of $NaOH$-Na_2CO_3-H_2O solutions.

Table S. 8. Composition and measured conductivity of Na_2CO_3+NaOH solutions at 277.65 K, 282.55 K, 293.55 K, and 308.45 K.

T/K	w_i/g		v_i/mL	m_i/mol·kg^{-1} (water)						κ/mS·cm^{-1} **
	H_2O	Na_2CO_3	NaOH	NaOH@*	Na^+	$Na(CO_3)^-$	OH^-	HCO_3^-	$[CO_3]^{2-}$	
			0	2.162×10^{-6}	9.227×10^{-3}	8.651×10^{-4}	4.338×10^{-4}	4.340×10^{-4}	3.747×10^{-3}	0.662
			0.050	9.102×10^{-6}	1.056×10^{-2}	1.010×10^{-3}	1.612×10^{-3}	1.203×10^{-4}	3.910×10^{-3}	0.836
			0.100	1.921×10^{-5}	1.194×10^{-2}	1.106×10^{-3}	3.040×10^{-3}	6.245×10^{-5}	3.864×10^{-3}	1.053
			0.150	3.143×10^{-5}	1.332×10^{-2}	1.190×10^{-3}	4.498×10^{-3}	4.107×10^{-5}	3.794×10^{-3}	1.274
277.65	33.2691	0.0178	0.250	6.162×10^{-5}	1.608×10^{-2}	1.338×10^{-3}	7.420×10^{-3}	2.352×10^{-5}	3.649×10^{-3}	1.710
			0.500	1.676×10^{-4}	2.296×10^{-2}	1.625×10^{-3}	1.465×10^{-2}	1.049×10^{-5}	3.337×10^{-3}	2.806
			0.750	3.131×10^{-4}	2.977×10^{-2}	1.838×10^{-3}	2.174×10^{-2}	6.343×10^{-6}	3.093×10^{-3}	3.853
			1.000	4.938×10^{-4}	3.649×10^{-2}	2.000×10^{-3}	2.869×10^{-2}	4.375×10^{-6}	2.896×10^{-3}	4.897
			0	2.504×10^{-6}	9.273×10^{-3}	7.076×10^{-4}	5.074×10^{-4}	5.078×10^{-4}	3.775×10^{-3}	0.752
			0.100	1.917×10^{-5}	1.201×10^{-2}	9.222×10^{-4}	3.065×10^{-3}	8.640×10^{-5}	3.969×10^{-3}	1.232
			0.250	6.099×10^{-5}	1.618×10^{-2}	1.128×10^{-3}	7.433×10^{-3}	3.315×10^{-5}	3.794×10^{-3}	1.948
282.55	33.2604	0.0176	0.501	1.663×10^{-4}	2.312×10^{-2}	1.388×10^{-3}	1.469×10^{-2}	1.494×10^{-5}	3.515×10^{-3}	3.168
			0.750	3.093×10^{-4}	2.992×10^{-2}	1.582×10^{-3}	2.175×10^{-2}	9.146×10^{-6}	3.291×10^{-3}	4.367
			1.000	4.875×10^{-4}	3.666×10^{-2}	1.735×10^{-3}	2.871×10^{-2}	6.356×10^{-6}	3.105×10^{-3}	5.520
			2.000	1.481×10^{-3}	6.255×10^{-2}	2.109×10^{-3}	5.524×10^{-2}	2.527×10^{-6}	2.598×10^{-3}	9.947
			0	3.469×10^{-6}	9.496×10^{-3}	4.847×10^{-4}	7.008×10^{-4}	7.013×10^{-4}	3.804×10^{-3}	1.043
			0.250	7.416×10^{-5}	1.650×10^{-2}	8.159×10^{-4}	7.468×10^{-3}	6.683×10^{-5}	4.073×10^{-3}	2.565
			0.500	1.650×10^{-4}	2.346×10^{-2}	1.023×10^{-3}	1.468×10^{-2}	3.102×10^{-5}	3.865×10^{-3}	4.099
293.55	33.2553	0.0176	0.750	3.072×10^{-4}	3.033×10^{-2}	1.184×10^{-3}	2.177×10^{-2}	1.931×10^{-5}	3.680×10^{-3}	5.599
			1.000	4.828×10^{-4}	3.709×10^{-2}	1.313×10^{-3}	2.872×10^{-2}	1.356×10^{-5}	3.520×10^{-3}	7.051
			2.000	1.461×10^{-3}	6.304×10^{-2}	1.649×10^{-3}	5.527×10^{-2}	5.593×10^{-6}	3.055×10^{-3}	12.522
			3.000	2.774×10^{-3}	8.730×10^{-2}	1.832×10^{-3}	7.997×10^{-2}	3.254×10^{-6}	2.745×10^{-3}	17.567
			0	5.326×10^{-6}	9.834×10^{-3}	3.097×10^{-4}	1.038×10^{-3}	1.039×10^{-3}	3.723×10^{-3}	1.504
			0.750	3.108×10^{-4}	3.083×10^{-2}	8.350×10^{-4}	2.178×10^{-2}	4.770×10^{-5}	4.081×10^{-3}	7.411
			1.000	4.869×10^{-4}	3.761×10^{-2}	9.359×10^{-4}	2.873×10^{-2}	3.405×10^{-5}	3.958×10^{-3}	9.325
308.45	33.2682	0.0179	2.000	1.465×10^{-3}	6.361×10^{-2}	1.210×10^{-3}	5.526×10^{-2}	1.455×10^{-5}	3.563×10^{-3}	16.486
			3.000	2.773×10^{-3}	8.788×10^{-2}	1.371×10^{-3}	7.995×10^{-2}	8.650×10^{-6}	3.276×10^{-3}	22.979
			4.000	4.315×10^{-3}	1.106×10^{-1}	1.473×10^{-3}	1.030×10^{-1}	5.934×10^{-6}	3.052×10^{-3}	28.976
			6.000	7.859×10^{-3}	1.519×10^{-1}	1.583×10^{-3}	1.449×10^{-1}	3.464×10^{-6}	2.714×10^{-3}	39.710

* Except the species listed above, other aqueous species exist in solution, including: $Na(HCO_3)$@, NaOH@, CO_2@, etc. Compared with the species in the table, the concentrations of these unlisted electrical neutrals are neglectable. Also, they don't affect the conductivity of electrolyte solutions.

** κ is the average of 4 repeatable measured values.

The standard uncertainty u(T) = 0.031 K, $u(w_i)$ = 0.00029 g, $u(v_i)$ = 0.14 μL. The combined standard uncertainty is shown in the following table. The expanded uncertainty of m_i varies with composition and temperature. For NaOH, $U(m_i)$≈2.5% with 99.5 % level of confidence (k≈2). For other species, $U(m_i)$≈5% with 99.73 % level of confidence (k=3).

Table S. 9. presents the uncertainties of the compositions and measured conductivities of NaOH-Na_2CO_3-H_2O solutions.

Table S. 9. Uncertainties of the composition and measured conductivity of Na_2CO_3+NaOH solutions at 277.65 K, 282.55 K, 293.55 K, and 308.45 K.

T/K	w_i/g		v_i/mL	$u^2_{c\text{-}2}(v_i)/mol^2 \cdot kg^{-2}$ (water)						$u(\kappa)/mS \cdot cm^{-1}$
	H_2O	Na_2CO_3	NaOH	NaOH@*	Na^+	$Na(CO_3)^-$	OH^-	HCO_3^-	$[CO_3]^{2-}$	
			0	2.260×10^{-15}	1.997×10^{-08}	5.380×10^{-10}	1.363×10^{-11}	1.255×10^{-11}	3.155×10^{-09}	0.000844
			0.050	1.335×10^{-14}	1.947×10^{-08}	6.070×10^{-10}	1.701×10^{-11}	2.425×10^{-12}	3.138×10^{-09}	0.000936
			0.100	4.115×10^{-14}	1.909×10^{-08}	6.618×10^{-10}	1.936×10^{-11}	7.804×10^{-13}	3.074×10^{-09}	0.000906
			0.150	9.379×10^{-14}	1.879×10^{-08}	6.992×10^{-10}	1.681×10^{-11}	3.088×10^{-13}	3.018×10^{-09}	0.000899
277.65	33.2691	0.0178	0.250	2.546×10^{-13}	1.822×10^{-08}	7.768×10^{-10}	1.850×10^{-11}	1.156×10^{-13}	2.859×10^{-09}	0.003924
			0.500	7.283×10^{-13}	1.701×10^{-08}	9.531×10^{-10}	1.492×10^{-11}	2.286×10^{-14}	2.506×10^{-09}	0.007041
			0.750	1.359×10^{-12}	1.600×10^{-08}	1.110×10^{-09}	1.566×10^{-11}	9.491×10^{-15}	2.216×10^{-09}	0.002614
			1.000	1.346×10^{-12}	1.522×10^{-08}	1.249×10^{-09}	1.484×10^{-11}	4.120×10^{-15}	1.981×10^{-09}	0.001683
			0	2.831×10^{-15}	2.097×10^{-08}	3.875×10^{-10}	1.771×10^{-11}	1.688×10^{-11}	3.485×10^{-09}	0.007307
			0.100	4.447×10^{-14}	2.012×10^{-08}	4.860×10^{-10}	1.700×10^{-11}	1.425×10^{-12}	3.462×10^{-09}	0.003008
			0.250	2.424×10^{-13}	1.929×10^{-08}	5.765×10^{-10}	1.560×10^{-11}	2.435×10^{-13}	3.270×10^{-09}	0.000882
282.55	33.2604	0.0176	0.501	5.871×10^{-13}	1.819×10^{-08}	7.059×10^{-10}	1.406×10^{-11}	5.233×10^{-14}	2.949×10^{-09}	0.001485
			0.750	1.553×10^{-12}	1.699×10^{-08}	8.635×10^{-10}	1.506×10^{-11}	1.962×10^{-14}	2.596×10^{-09}	0.004960
			1.000	2.159×10^{-12}	1.623×10^{-08}	9.604×10^{-10}	1.656×10^{-11}	9.561×10^{-15}	2.383×10^{-09}	0.002912
			2.000	5.020×10^{-12}	1.364×10^{-08}	1.312×10^{-09}	1.361×10^{-11}	1.586×10^{-15}	1.712×10^{-09}	0.001842
			0	5.997×10^{-15}	2.267×10^{-08}	1.933×10^{-10}	3.045×10^{-11}	3.153×10^{-11}	3.153×10^{-11}	0.001237
			0.250	2.257×10^{-13}	2.107×10^{-08}	3.159×10^{-10}	1.568×10^{-11}	9.109×10^{-13}	9.109×10^{-13}	0.002096
			0.500	7.354×10^{-13}	1.990×10^{-08}	4.117×10^{-10}	1.392×10^{-11}	2.063×10^{-13}	2.063×10^{-13}	0.003527
293.55	33.2553	0.0176	0.750	9.846×10^{-13}	1.882×10^{-08}	5.210×10^{-10}	1.419×10^{-11}	6.098×10^{-14}	6.098×10^{-14}	0.002538
			1.000	2.300×10^{-12}	1.814×10^{-08}	5.605×10^{-10}	1.562×10^{-11}	4.538×10^{-14}	4.538×10^{-14}	0.002257
			2.000	7.314×10^{-12}	1.538×10^{-08}	8.175×10^{-10}	1.470×10^{-11}	8.730×10^{-15}	8.730×10^{-15}	0.005767
			3.000	7.481×10^{-12}	1.373×10^{-08}	9.688×10^{-10}	1.426×10^{-11}	2.710×10^{-15}	2.710×10^{-15}	0.006425
			0	1.398×10^{-14}	2.395×10^{-08}	9.168×10^{-11}	7.020×10^{-11}	6.838×10^{-11}	4.171×10^{-09}	0.002340
			0.750	1.158×10^{-12}	2.103×10^{-08}	2.216×10^{-10}	1.429×10^{-11}	5.057×10^{-13}	4.212×10^{-09}	0.013279
			1.000	2.642×10^{-12}	2.014×10^{-08}	2.635×10^{-10}	1.265×10^{-11}	2.630×10^{-13}	3.987×10^{-09}	0.001996
308.45	33.2682	0.0179	2.000	4.096×10^{-12}	1.749×10^{-08}	4.388×10^{-10}	1.278×10^{-11}	4.672×10^{-14}	3.187×10^{-09}	0.003698
			3.000	9.170×10^{-12}	1.559×10^{-08}	5.302×10^{-10}	1.572×10^{-11}	1.822×10^{-14}	2.736×10^{-09}	0.014048
			4.000	1.013×10^{-11}	1.433×10^{-08}	5.750×10^{-10}	1.455×10^{-11}	9.463×10^{-15}	2.439×10^{-09}	0.007953
			6.000	1.656×10^{-11}	1.198×10^{-08}	6.724×10^{-10}	1.827×10^{-11}	3.113×10^{-15}	1.913×10^{-09}	0.020304

7. Composition and conductivity of NaOH-Na_2SiO_3-H_2O solutions at different temperatures

Table S. 10. presents the compositions and measured conductivities of NaOH-Na_2SiO_3-H_2O solutions.

Table S. 10. Composition and measured conductivity of $NaOH$-Na_2SiO_3-H_2O solutions at 277.85 K, 282.65 K, 287.65 K,293.65 K, 298.55 K, 303.45 K, and 308.45 K.

No.	T/K	w_i/g		v_i/mL	m_i/mol·kg^{-1} (water)							κ/mS·cm^{-1}*
		H_2O	$Na_2SiO_3\cdot 5H_2O$	NaOH	NaOH@	SiO_2@	Na^+	OH^-	$[SiO(OH)_3]^-$	$[SiO_2(OH)_2]^{2-}$	$[Si_4O_{10}]^{4-}$	
S1	277.85	33.2713	0.0352	0	2.634×10^{-5}	2.892×10^{-5}	0.0099479	0.0047875	0.0047554	0.0002022	1.399×10^{-7}	0.967
				0.005	3.865×10^{-5}	2.204×10^{-5}	0.0114211	0.0062013	0.0046948	0.0002622	1.443×10^{-7}	1.195
				0.100	5.289×10^{-5}	1.770×10^{-5}	0.0128880	0.0076119	0.0046322	0.0003216	1.475×10^{-7}	1.422
				0.150	6.898×10^{-5}	1.474×10^{-5}	0.0143485	0.0090179	0.0045687	0.0003807	1.498×10^{-7}	1.659
				0.250	1.066×10^{-4}	1.093×10^{-5}	0.0172510	0.0118165	0.0044428	0.0004956	1.522×10^{-7}	2.099
				0.500	2.291×10^{-4}	6.419×10^{-6}	0.0244025	0.0187300	0.0041405	0.0007657	1.498×10^{-7}	3.213
				0.750	3.886×10^{-4}	4.387×10^{-6}	0.0314099	0.0255276	0.0038624	0.0010097	1.406×10^{-7}	4.290
				1.000	5.809×10^{-4}	3.245×10^{-6}	0.0382799	0.0322135	0.0036095	0.0012282	1.286×10^{-7}	5.342
S2	282.65	33.0836	0.0352	0	2.639×10^{-5}	3.519×10^{-5}	0.0100044	0.0048435	0.0047985	0.0001807	2.535×10^{-7}	1.085
				0.100	5.295×10^{-5}	2.163×10^{-5}	0.0129611	0.0076944	0.0046894	0.0002881	2.711×10^{-7}	1.585
				0.250	1.066×10^{-4}	1.342×10^{-5}	0.0173487	0.0119386	0.0045174	0.0004458	2.850×10^{-7}	2.348
				0.500	2.290×10^{-4}	7.937×10^{-6}	0.0245403	0.0189143	0.0042383	0.0006932	2.887×10^{-7}	3.577
				0.750	3.882×10^{-4}	5.460×10^{-6}	0.0315868	0.0257684	0.0039778	0.0009197	2.781×10^{-7}	4.786
				1.000	5.801×10^{-4}	4.062×10^{-6}	0.0384948	0.0325058	0.0037381	0.0011249	2.604×10^{-7}	5.939
S3	287.65	33.1705	0.0352	0	2.614×10^{-5}	4.264×10^{-5}	0.0099784	0.0048572	0.0047962	0.0001616	4.528×10^{-7}	1.270
				0.100	5.240×10^{-5}	2.631×10^{-5}	0.0129277	0.0077085	0.0047007	0.0002582	4.902×10^{-7}	1.818
				0.250	1.055×10^{-4}	1.640×10^{-5}	0.0173045	0.0119552	0.0045454	0.0004009	5.236×10^{-7}	2.647
				0.500	2.264×10^{-4}	9.794×10^{-6}	0.0244789	0.0189349	0.0042900	0.0006259	5.505×10^{-7}	4.000
				0.750	3.836×10^{-4}	6.775×10^{-6}	0.0315095	0.0257900	0.0040479	0.0008347	5.422×10^{-7}	5.316
				1.000	5.731×10^{-4}	5.066×10^{-6}	0.0384025	0.0325258	0.0038226	0.0010260	5.184×10^{-7}	6.591
				2.000	1.595×10^{-3}	2.264×10^{-6}	0.0647026	0.0583558	0.0030823	0.0016315	3.784×10^{-7}	11.438
S4	293.65	33.2451	0.0352	0	2.601×10^{-5}	5.358×10^{-5}	0.0099561	0.0048741	0.0047891	0.0001446	9.467×10^{-7}	1.383
				0.250	1.046×10^{-4}	2.070×10^{-5}	0.0172666	0.0119726	0.0045676	0.0003610	1.112×10^{-6}	2.810
				0.500	2.246×10^{-4}	1.238×10^{-5}	0.0244262	0.0189541	0.0043325	0.0005674	1.180×10^{-6}	4.357
				0.750	3.805×10^{-4}	8.608×10^{-6}	0.0314426	0.0258101	0.0041071	0.0007603	1.186×10^{-6}	5.854
				1.000	5.682×10^{-4}	6.490×10^{-6}	0.0383225	0.0325464	0.0038965	0.0009375	1.171×10^{-6}	7.317
				2.000	1.580×10^{-3}	2.944×10^{-6}	0.0645775	0.0583599	0.0031881	0.0015129	9.105×10^{-7}	12.759
S5	298.55	33.2514	0.0352	0	2.606×10^{-5}	6.343×10^{-5}	0.0099541	0.0048933	0.0047857	0.0001342	1.691×10^{-6}	1.524
				0.250	1.046×10^{-4}	2.475×10^{-5}	0.0172634	0.0119997	0.0045843	0.0003356	2.050×10^{-6}	3.212
				0.500	2.245×10^{-4}	1.486×10^{-5}	0.0244217	0.0189911	0.0043631	0.0005293	2.210×10^{-6}	4.952
				0.750	3.801×10^{-4}	1.036×10^{-5}	0.0314372	0.0258561	0.0041491	0.0007115	2.252×10^{-6}	6.588
				1.000	5.675×10^{-4}	7.807×10^{-6}	0.0383161	0.0325986	0.0039466	0.0008810	2.222×10^{-6}	8.174
				2.000	1.577×10^{-3}	3.576×10^{-6}	0.0645691	0.0584300	0.0032601	0.0014359	1.803×10^{-6}	14.136
S6	303.45	32.7884	0.0352	0	2.687×10^{-5}	7.558×10^{-5}	0.0100943	0.0049776	0.0048397	0.0001317	3.396×10^{-6}	1.696
				0.500	2.305×10^{-4}	1.781×10^{-5}	0.0247584	0.0192711	0.0044279	0.0005206	4.536×10^{-6}	5.456
				0.750	3.901×10^{-4}	1.244×10^{-5}	0.0318665	0.0262308	0.0042154	0.0007008	4.657×10^{-6}	7.217
				1.000	5.820×10^{-4}	9.379×10^{-6}	0.0388344	0.0330642	0.0040138	0.0008689	4.624×10^{-6}	8.978
				2.000	1.614×10^{-3}	4.315×10^{-6}	0.0654121	0.0592251	0.0033278	0.0014220	3.829×10^{-6}	15.493
				3.000	2.969×10^{-3}	2.561×10^{-6}	0.0901232	0.0836745	0.0028069	0.0018151	2.868×10^{-6}	21.422

				4.000	4.555×10^{-3}	1.716×10^{-6}	0.1131860	0.1065856	0.0024078	0.0020921	2.098×10^{-6}	26.892
S7	308.45	33.2020	0.0352	0	2.643×10^{-5}	8.906×10^{-5}	0.0099686	0.0049370	0.0047615	0.0001231	5.968×10^{-6}	1.860
				0.750	3.912×10^{-4}	1.478×10^{-5}	0.0318654	0.0262580	0.0042220	0.0006748	8.916×10^{-6}	7.912
				1.000	5.835×10^{-4}	1.116×10^{-5}	0.0388330	0.0330955	0.0040259	0.0008380	8.917×10^{-6}	9.676
				2.000	1.617×10^{-3}	5.162×10^{-6}	0.0654094	0.0592660	0.0033549	0.0013791	7.568×10^{-6}	16.756
				3.000	2.973×10^{-3}	3.078×10^{-6}	0.0901198	0.0837184	0.0028415	0.0017683	5.781×10^{-6}	23.260
				4.000	4.559×10^{-3}	2.068×10^{-6}	0.1131827	0.1066294	0.0024453	0.0020454	4.295×10^{-6}	29.222
S0	298.55	33.2570	0.0142	0	4.736×10^{-6}	6.126×10^{-5}	0.0040209	0.0020483	0.0019304	0.0000210	2.869×10^{-8}	0.610
			0.0394		3.209×10^{-5}	6.359×10^{-5}	0.0111347	0.0054463	0.0053400	0.0001686	2.806×10^{-6}	1.687
			0.063		7.578×10^{-4}	6.368×10^{-5}	0.0177753	0.0084801	0.0083361	0.0004333	2.311×10^{-5}	2.655
			0.0838		1.263×10^{-4}	6.281×10^{-5}	0.0236132	0.0110497	0.0107286	0.0007566	8.044×10^{-5}	3.484
			0.1081		1.977×10^{-4}	6.068×10^{-5}	0.0304177	0.0139529	0.0131098	0.0012178	2.298×10^{-4}	4.442

* κ is the average of 4 repeatable measured values.

The standard uncertainty u(T) = 0.031 K, $u(w_i)$ = 0.00029 g, $u(v_i)$ = 0.14 µL. The combined standard uncertainty is shown in the following table. The expanded uncertainty of m_i varies with composition, temperature and species. In general, k≈3 leads to an approximate level of confidence of 99.73%. For each species, the expanded uncertainty is presented by its maximum of all calculated value under different temperatures and compositions. Thus, $U(m_{NaOH@})$=4.62 %, $U(m_{SiO_2@})$=2.49 %, $U(m_{Na^+})$=2.97 %, $U(m_{OH^-})$=2.50 %, $U(m_{[SiO(OH)_3]^-})$=4.20 %, $U(m_{[SiO_2(OH)_2]^{2-}})$=5.23 %, and $U(m_{[Si_4O_{10}]^{4-}})$=16.62 %.

Table S. 11. presents the uncertainties of the compositions and measured conductivities of $NaOH$-Na_2SiO_3-H_2O solutions.

Table S. 11. Uncertainties of the composition and measured conductivity of $NaOH$-Na_2SiO_3-H_2O solutions at 277.85 K, 282.65 K, 287.65 K,293.65 K, 298.55 K, 303.45 K, and 308.45 K.

No.	T/K	w_i/g		v_i/mL	$u^2_{m_i}$ /mol·kg^{-1} (water)							u(κ)/mS·cm^{-1}
		H_2O	$Na_2SiO_3\cdot5H_2O$	NaOH	NaOH@	SiO_2@	Na^+	OH^-	$[SiO(OH)_3]^-$	$[SiO_2(OH)_2]^{2-}$	$[Si_4O_{10}]^{4-}$	
S1	277.85	33.2713	0.0352	0	1.631×10^{-13}	2.048×10^{-15}	6.693×10^{-9}	1.407×10^{-9}	1.420×10^{-9}	1.131×10^{-11}	2.697×10^{-17}	0.001837
				0.005	2.304×10^{-13}	2.940×10^{-15}	6.659×10^{-9}	1.364×10^{-9}	1.376×10^{-9}	1.477×10^{-11}	2.800×10^{-17}	0.001795
				0.100	3.055×10^{-13}	3.560×10^{-15}	6.628×10^{-9}	1.343×10^{-9}	1.359×10^{-9}	1.635×10^{-11}	2.872×10^{-17}	0.001864
				0.150	3.871×10^{-13}	3.761×10^{-15}	6.595×10^{-9}	1.280×10^{-9}	1.299×10^{-9}	2.269×10^{-11}	2.918×10^{-17}	0.000422
				0.250	7.667×10^{-13}	3.106×10^{-15}	6.515×10^{-9}	1.189×10^{-9}	1.226×10^{-9}	3.231×10^{-11}	2.944×10^{-17}	0.003959
				0.500	1.051×10^{-12}	1.660×10^{-15}	6.389×10^{-9}	1.021×10^{-9}	1.069×10^{-9}	5.966×10^{-11}	2.751×10^{-17}	0.001392
				0.750	1.616×10^{-12}	9.173×10^{-16}	6.252×10^{-9}	8.741×10^{-10}	9.335×10^{-10}	9.190×10^{-11}	2.382×10^{-17}	0.002030
				1.000	2.408×10^{-13}	5.408×10^{-16}	6.112×10^{-9}	7.408×10^{-10}	8.148×10^{-10}	1.279×10^{-10}	1.981×10^{-17}	0.000960
S2	282.65	33.0836	0.0352	0	1.643×10^{-13}	3.809×10^{-15}	6.771×10^{-9}	1.451×10^{-9}	1.462×10^{-9}	9.105×10^{-12}	8.961×10^{-17}	0.002485
				0.100	3.080×10^{-13}	5.343×10^{-15}	6.703×10^{-9}	1.370×10^{-9}	1.383×10^{-9}	1.512×10^{-11}	9.793×10^{-17}	0.000406
				0.250	5.880×10^{-13}	4.686×10^{-15}	6.607×10^{-9}	1.250×10^{-9}	1.278×10^{-9}	2.643×10^{-11}	1.041×10^{-16}	0.001796
				0.500	1.106×10^{-12}	2.506×10^{-15}	6.456×10^{-9}	1.078×10^{-9}	1.126×10^{-9}	4.976×10^{-11}	1.032×10^{-16}	0.001451
				0.750	1.708×10^{-12}	1.416×10^{-15}	6.316×10^{-9}	9.318×10^{-10}	9.947×10^{-10}	7.735×10^{-11}	9.359×10^{-17}	0.002704
				1.000	2.169×10^{-12}	8.584×10^{-16}	6.193×10^{-9}	8.121×10^{-10}	8.829×10^{-10}	1.067×10^{-10}	8.099×10^{-17}	0.001671
S3	287.65	33.1705	0.0352	0	1.604×10^{-13}	1.252×10^{-13}	6.736×10^{-9}	1.509×10^{-9}	1.485×10^{-9}	5.975×10^{-12}	6.276×10^{-16}	0.001639
				0.100	3.033×10^{-13}	7.916×10^{-15}	6.667×10^{-9}	1.392×10^{-9}	1.400×10^{-9}	1.222×10^{-11}	3.226×10^{-16}	0.001929
				0.250	7.355×10^{-13}	7.052×10^{-15}	6.558×10^{-9}	1.274×10^{-9}	1.304×10^{-9}	2.129×10^{-11}	3.535×10^{-16}	0.003691
				0.500	9.594×10^{-12}	4.040×10^{-15}	3.706×10^{-8}	1.819×10^{-8}	1.898×10^{-9}	1.187×10^{-10}	7.930×10^{-16}	0.002731
				0.750	4.107×10^{-11}	1.526×10^{-15}	9.698×10^{-8}	4.605×10^{-8}	3.214×10^{-9}	4.002×10^{-10}	1.313×10^{-15}	0.002527
				1.000	2.663×10^{-12}	1.276×10^{-15}	6.139×10^{-9}	8.388×10^{-10}	9.183×10^{-10}	9.179×10^{-11}	3.256×10^{-16}	0.000713

				2.000	5.068×10^{-12}	3.026×10^{-16}	5.667×10^{-9}	5.095×10^{-10}	6.064×10^{-10}	2.007×10^{-10}	1.676×10^{-16}	0.004666
S4	293.65	33.2451	0.0352	0	1.601×10^{-13}	6.735×10^{-15}	6.703×10^{-9}	1.483×10^{-9}	1.480×10^{-9}	5.921×10^{-12}	1.243×10^{-15}	0.000664
				0.250	5.609×10^{-13}	1.117×10^{-14}	6.553×10^{-9}	1.311×10^{-9}	1.323×10^{-9}	1.737×10^{-11}	1.602×10^{-15}	0.002079
				0.500	1.095×10^{-12}	5.990×10^{-15}	6.407×10^{-9}	1.150×10^{-9}	1.186×10^{-9}	3.393×10^{-11}	1.740×10^{-15}	0.003402
				0.750	1.730×10^{-12}	3.409×10^{-15}	6.269×10^{-9}	1.012×10^{-9}	1.065×10^{-9}	5.402×10^{-11}	1.723×10^{-15}	0.001277
				1.000	2.193×10^{-12}	2.148×10^{-15}	6.151×10^{-9}	9.028×10^{-10}	9.632×10^{-10}	7.526×10^{-11}	1.647×10^{-15}	0.004083
				2.000	4.406×10^{-12}	5.145×10^{-16}	5.705×10^{-9}	5.664×10^{-10}	6.515×10^{-10}	1.720×10^{-10}	9.728×10^{-16}	0.005264
S5	298.55	33.2514	0.0352	0	1.607×10^{-13}	4.993×10^{-15}	6.703×10^{-9}	1.493×10^{-9}	1.487×10^{-9}	5.112×10^{-12}	4.055×10^{-15}	0.007123
				0.250	5.624×10^{-13}	1.466×10^{-14}	6.542×10^{-9}	1.327×10^{-9}	1.328×10^{-9}	1.548×10^{-11}	5.401×10^{-15}	0.008832
				0.500	1.075×10^{-12}	8.457×10^{-15}	6.395×10^{-9}	1.177×10^{-9}	1.202×10^{-9}	2.940×10^{-11}	5.996×10^{-15}	0.002765
				0.750	1.643×10^{-12}	4.853×10^{-15}	6.258×10^{-9}	1.043×10^{-9}	1.087×10^{-9}	4.694×10^{-11}	6.112×10^{-15}	0.001245
				1.000	2.187×10^{-12}	3.033×10^{-15}	6.130×10^{-9}	9.294×10^{-10}	9.855×10^{-10}	6.662×10^{-11}	5.854×10^{-15}	0.003301
				2.000	4.989×10^{-12}	7.317×10^{-16}	5.655×10^{-9}	5.833×10^{-10}	6.765×10^{-10}	1.566×10^{-10}	3.751×10^{-15}	0.003868
S6	303.45	32.7884	0.0352	0	1.702×10^{-13}	2.268×10^{-14}	6.893×10^{-9}	1.541×10^{-9}	1.515×10^{-9}	4.906×10^{-12}	1.651×10^{-14}	0.005074
				0.500	1.147×10^{-12}	1.276×10^{-14}	6.569×10^{-9}	1.220×10^{-9}	1.223×10^{-9}	2.832×10^{-11}	2.554×10^{-14}	0.002235
				0.750	1.749×10^{-12}	7.052×10^{-15}	6.427×10^{-9}	1.085×10^{-9}	1.108×10^{-9}	4.516×10^{-11}	2.620×10^{-14}	0.009191
				1.000	2.223×10^{-12}	4.550×10^{-15}	6.301×10^{-9}	9.717×10^{-10}	1.008×10^{-9}	6.366×10^{-11}	2.539×10^{-14}	0.001087
				2.000	5.410×10^{-12}	1.056×10^{-15}	5.796×10^{-9}	6.126×10^{-10}	6.974×10^{-10}	1.516×10^{-10}	1.690×10^{-14}	0.007467
				3.000	6.700×10^{-12}	4.023×10^{-16}	5.415×10^{-9}	4.132×10^{-10}	5.032×10^{-10}	2.352×10^{-10}	9.433×10^{-15}	0.003547
				4.000	8.202×10^{-12}	1.876×10^{-16}	5.066×10^{-9}	2.788×10^{-10}	3.741×10^{-10}	3.062×10^{-10}	5.063×10^{-15}	0.005527
S7	308.45	33.2020	0.0352	0	1.643×10^{-13}	2.676×10^{-14}	6.722×10^{-9}	1.512×10^{-9}	1.457×10^{-9}	4.273×10^{-12}	5.082×10^{-14}	0.000940
				0.750	1.809×10^{-12}	9.402×10^{-15}	6.266×10^{-9}	1.077×10^{-9}	1.067×10^{-9}	3.995×10^{-11}	8.537×10^{-14}	0.002294
				1.000	2.256×10^{-12}	6.094×10^{-15}	6.144×10^{-9}	9.714×10^{-10}	9.748×10^{-10}	5.616×10^{-11}	8.376×10^{-14}	0.003065
				2.000	5.008×10^{-12}	1.503×10^{-15}	5.670×10^{-9}	6.295×10^{-10}	6.864×10^{-10}	1.346×10^{-10}	5.955×10^{-14}	0.011025
				3.000	6.821×10^{-12}	5.760×10^{-16}	5.284×10^{-9}	4.251×10^{-10}	5.017×10^{-10}	2.122×10^{-10}	3.502×10^{-14}	0.007165
				4.000	7.823×10^{-12}	2.705×10^{-16}	4.953×10^{-9}	2.963×10^{-10}	3.775×10^{-10}	2.796×10^{-10}	1.950×10^{-14}	0.008813
S0	298.55	33.2570	0.0142	0	3.412×10^{-13}	6.360×10^{-15}	6.727×10^{-9}	1.608×10^{-9}	1.610×10^{-9}	7.869×10^{-13}	7.370×10^{-18}	0.000437
			0.0394		1.918×10^{-13}	1.501×10^{-15}	6.678×10^{-9}	1.454×10^{-9}	1.457×10^{-9}	6.401×10^{-12}	8.927×10^{-15}	0.005296
			0.063		3.978×10^{-13}	9.568×10^{-15}	6.639×10^{-9}	1.331×10^{-9}	1.234×10^{-9}	1.599×10^{-11}	2.292×10^{-13}	0.003284
			0.0838		6.231×10^{-13}	2.890×10^{-15}	6.646×10^{-9}	1.243×10^{-9}	9.777×10^{-10}	2.596×10^{-11}	1.508×10^{-12}	0.003213
			0.1081		8.447×10^{-13}	2.476×10^{-15}	6.581×10^{-9}	1.168×10^{-9}	6.499×10^{-10}	3.623×10^{-11}	5.908×10^{-12}	0.001677

The standard uncertainty u(κ) is calculated via Type A evaluation [28]. The maximum of u(κ) is 0.011025 $mS \cdot cm^{-1}$.

Figure S. 10. Shows the mean square variance (MSV) of R, ranging from 0.9 to 1.7, when solving the equations in group S0. Figure S. 11 addresses MSV and the maximum deviation of MSV when R ranges from 0.9 to 1.7 in solving the equations in groups S1 to S6. The data of group S7, measured at 308.45 K, is not introduced into the regression analysis, as the deviation at this temperature is so comparably high that it will deteriorate the global statistical significance.

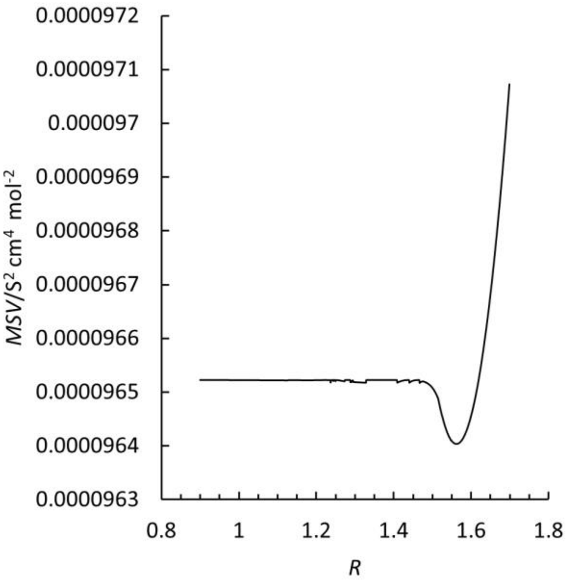

Figure S. 10. R dependence of mean square variance, MSV, of the equivalent conductivity of $[SiO(OH)_3]^-$ and $[SiO_2(OH)_2]^{2-}$ ions of group S0 at 298.55 K.

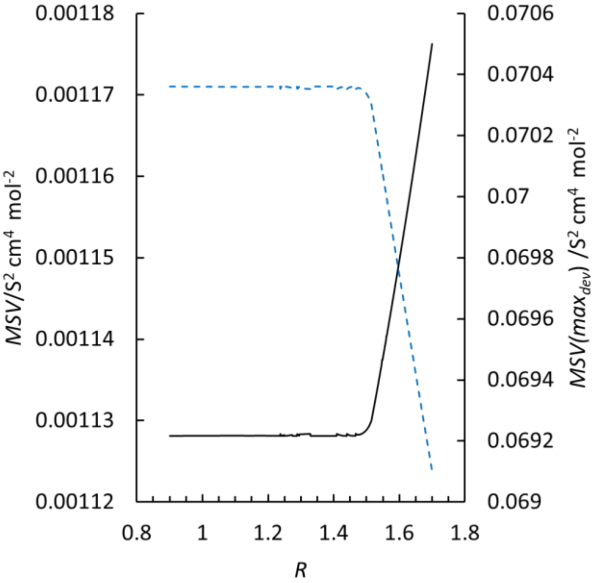

Figure S. 11. R dependence of averaged mean square variance, (MSV) and MSV of the maximum deviation (dev) of the equivalent conductivity of $[SiO(OH)_3]^-$ and $[SiO_2(OH)_2]^{2-}$ ions of group S1 to S6.

Figure S. 12. presents the conductivity of water saturated with atmospheric CO_2 at different temperatures [12].

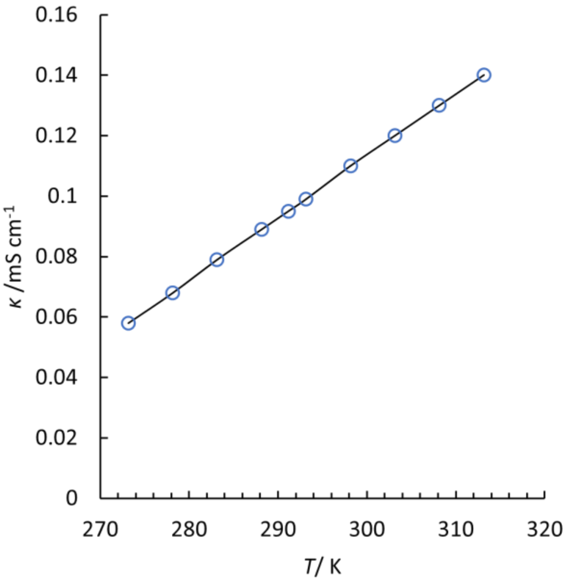

Figure S. 12. Conductivity of water saturated with atmospheric CO_2 at different temperatures.
